# New sesquiterpenoids with anti-inflammatory effects from phytopathogenic fungus *Bipolaris sorokiniana* 11134

**DOI:** 10.1007/s13659-025-00508-9

**Published:** 2025-05-09

**Authors:** Qiang Yin, Jianying Han, Guixiang Yang, Zhijun Song, Keke Zou, Kangjie Lv, Zexu Lin, Lei Ma, Miaomiao Liu, Yunjiang Feng, Ronald J. Quinn, Tom Hsiang, Lixin Zhang, Xueting Liu, Guoliang Zhu, Jingyu Zhang

**Affiliations:** 1 State Key Laboratory of Bioreactor Engineering, East China University of Science and Technology, Shanghai, 200237 China; 2 Griffith Institute for Drug Discovery, Griffith University, Brisbane, QLD Australia; 3 Institute for Molecular Bioscience, The University of Queensland, St. Lucia, QLD 4072 Australia; 4 Chinese Academy of Sciences, Key Laboratory of Pathogenic Microbiology and Immunology, Institute of Microbiology, Chinese Academy of Sciences, Beijing, China; 5 School of Environmental Sciences, University of Guelph, Guelph, ON N1G 2W1 Canada

**Keywords:** Phytopathogenic fungi, *Bipolaris sorokiniana* 11134, Sesquiterpenoid, Anti-inflammatory

## Abstract

Sesquiterpenoids represent a structurally diverse class of natural products widely recognized for their ecological significance and pharmacological potential, including antimicrobial, anti-inflammatory, and anticancer properties. As part of our efforts to explore bioactive secondary metabolites from phytopathogenic fungi, we conducted a molecular networking-based analysis of *Bipolaris sorokiniana* isolate BS11134, which was fermented on a rice medium. This analysis led to the identification of three new seco-sativene-type sesquiterpenoids (**1**–**3**) and seven known analogues (**4**–**10**), with the NMR data of compound **4** being reported for the first time. The structures of these compounds were elucidated using HR-ESI-MS and extensive spectroscopic data analysis. Notably, compound **9** significantly inhibited nitrous oxide expression in lipopolysaccharide (LPS)-treated RAW264.7 cells in vitro (inhibition rate: 84.7 ± 1.7% at 10 μM), while compound **1** (10 μM) showed a weak inhibitory effect (inhibition rate = 28.0 ± 2.4%). Additionally, we proposed a biosynthetic pathway for these compounds. This study not only expands the chemical space of the helminthoporene class of molecules but also underscores the untapped potential of phytopathogenic fungi as promising sources of structurally unique and biologically active natural products.

**Graphical Abstract:**

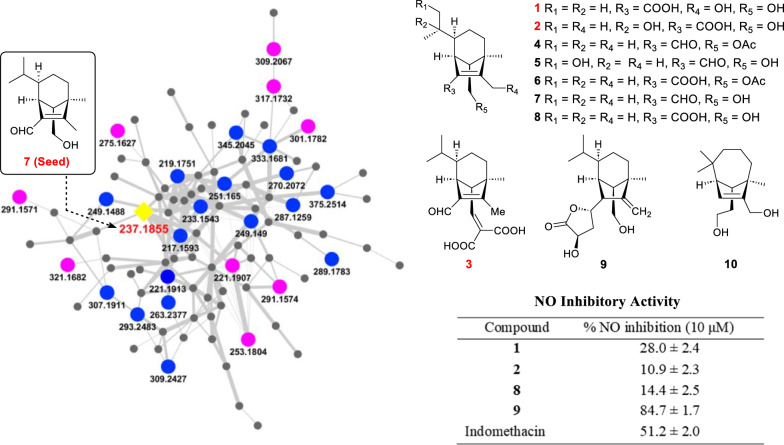

**Supplementary Information:**

The online version contains supplementary material available at 10.1007/s13659-025-00508-9.

## Introduction

Inflammation in animal tissues is a complex reaction regulated by several inflammatory mediators, including nitric oxide (NO), prostaglandins E2, cytokines, and growth factors [1]. Inflammation is commonly associated with various pathophysiological conditions, such as arthritis, tumours, Alzheimer, or cardiovascular diseases [2–4]. Macrophages, pivotal in host defence, release inflammatory mediators upon activation by lipopolysaccharides (LPS), contributing to pathogenesis. Thus, inhibition of macrophage-produced mediators may attenuate the development of related diseases.

Sesquiterpenes play significant roles in biology and ecology [5], displaying a range of activities in fighting against tumors, viruses, fungi, HIV, biotic pressures, immunity issues, and pests [6–13].

Fungi, especially those interacting with plants, are a rich source of novel sesquiterpenes [14, 15]. Based on cross-talk between plants and phytopathogens, phytopathogenic fungi can co-evolve with host plants, which leads to the production of unique natural products. Phytopathogenic fungi are a source for the discovery of new active compounds.

Traditionally, the isolation of secondary metabolite (SMs) has relied on bioactivity-directed isolation or via screening targets with distinct spectroscopic features. Recently, Molecular Networking (MN) has revolutionized the visualization of chemical diversity of extracts through the spectral alignment of tandem mass spectrometry (MS^b^) data, which captures in-source fragmentation and ionization patterns [16, 17]. MN is particularly adept at identifying unclassified molecules that may hold biological significance or represent novel metabolic pathways, especially useful in comparative studies of different states, such as time points or genetic variant [18]. To enhance the capabilities of MN, Feature-based molecular networking (FBMN) was introduced in 2020, offering enhanced functionality by incorporating chromatographic feature detection into spectral alignment [19].

*Bipolaris sorokiniana*, a well-known phytopathogenic fungus, causes diseases of barley and wheat, such as root rots, leaf spots, seedling blight, and head blight [20]. Known for producing a range of phytotoxic substance [21, 22], this fungus has been linked to approximately 20 sesquiterpenoids related to helminthosporol across different *Bipolaris* species. In this study, we report the discovery of ten sesquiterpenoids (**1**–**10**) from *B. sorokiniana* with a helminthoporene skeleton. This included the identification of three new *seco*-sativene type compounds (**1**–**3**) through Global Natural Product Social (GNPS) molecular networking and LC–MS/MS analysis. Additionally, we assessed the anti-inflammatory potential of these isolated sesquiterpenoids in vitro, focusing on their capacity to inhibit NO production induced by LPS in the cell lines of RAW264.7.

## Results

### Sesquiterpenoids gene clusters exploration from BS11134 based on genome analysis

**Fig. 1:**
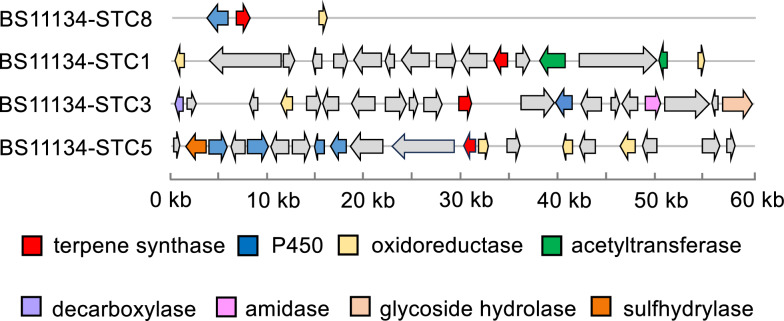
Organization of the sesquiterpenoids biosynthetic gene cluster in BS11134

BS11134 was classified as *Bipolaris sorokiniana* based on morphology and sequence of the internal transcribed spacer (ITS) region (GenBank accession number KU297882) [23]. We used the Hidden Markov Models file (Terpene_syn_C_2) classified in the Pfam database [24] to examine the BS11134 genome obtained in our previous study [23] for potential sesquiterpene cyclase (STC) genes, resulting in four homologous sequences of known STC proteins with DDXXD metal binding motifs. Further annotation of these four STC-encoding biosynthetic gene clusters using antiSMAS [25] and 2ndfinder online software revealed various post-modification enzymes, including cytochrome P450, acetyltransferase, decarboxylase, etc. (Fig. 1). Notably, the length of STC8 gene cluster was only 15 kb (Fig. 1), consisting of a three-gene cassette very similar to the recently identified *seco*-sativene gene cluste [26].

### FBMN guided detection of sesquiterpenoids

**Fig. 2:**
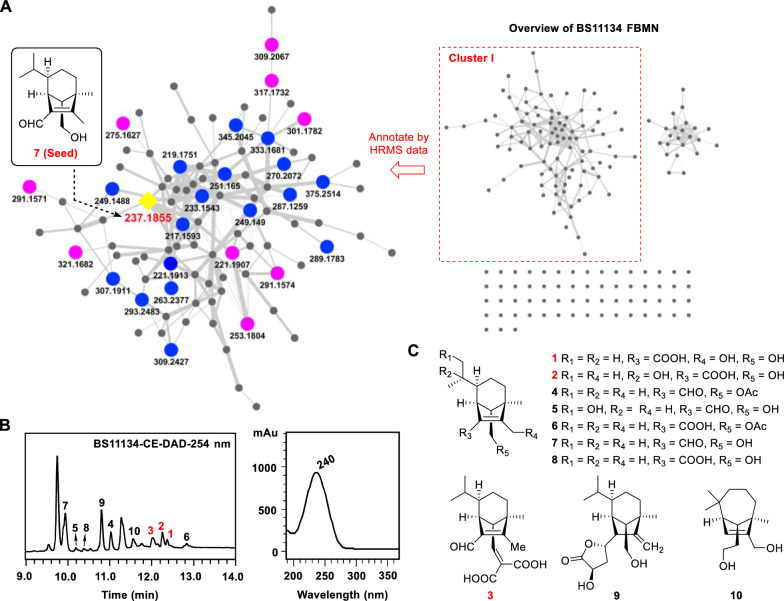
FBMN-guided characterization of new helminthosporol analogs from BS11134 crude extract. **A** Clustering of sesquiterpenoids analogs. Helminthosporol (**7**) was used as seed compound (yellow shaded square) in cluster analysis, and 26 analogs (Purple circles: compounds isolated in this study; Blue circles: other potential new sesquiterpenoids identified by HRMS annotation) were identified within the network. **B** HPLC–UV chromatogram of sesquiterpenoids fraction RD3 examined at 254 nm, and the UV spectrum of helminthosporol. **C** Structures of compounds **1**–**10** isolated from strain BS11134

We used rice medium to ferment the fungus because this medium gave the most induced secondary metabolites compared with other six media used [23]. A previously reported *seco*-sativene type sesquiterpenoid helminthosporol (**7**) was characterized from the crude extract of BS11134 based on its UV spectrum and HR-MS data (Fig. 2B and Fig. S7a). Further analysis using GNPS FBMN identified new *seco*-sativene type sesquiterpenoids using helminthosporol (**7**) as the seed compound (Fig. 2A), the precursor m/z of **7** was found to be within Cluster I, comprising 97 nodes with similar MS/MS patterns.

Detailed HRESIMS data annotation (Table 1) revealed 27 potential sesquiterpenoids, ten of which showed similar UV spectra to helminthosporol in the HPLC chromatogram of subfraction RD3. Among these, five nodes exhibited previously unreported *m*/*z* values (291.1571, 291.1574, 321.1682, 301.1782, 253.1804), guiding the isolation of new sesquiterpenoids from several subfractions. Three new sesquiterpenoids (**1**–**3**) with a helminthoporene skeleton as well as seven known compounds (**4**–**10**) were identified (Fig. 2C).

**Table 1:** Detailed annotation for sesquiterpnoid related nodes in cluster I from the BS11134 featured based molecular networking

Node (observed precursor *m*/*z*)	Molecular formula	Calc. *m/z*	^a^Δ (ppm)	Δ_m/z_	Annotation
217.1593	C_15_H_21_O	217.1587	2.8	− 4.0320 from **10**	− H_4_ from **10**
219.1751	C_15_H_23_O	219.1743	3.7	− 2.0156 from **10**	− H_2_ from **10**
221.1907	C_15_H_25_O	221.1900	3.2	− 15.9948 from **7**	Secolongifolene diol (**10**) [M−H_2_O+H]^+^
221.1913	C_15_H_25_O	221.1900	5.8	− 15.9942 from **7**	Isomer of **10**
233.1543	C_15_H_21_O_2_	233.1536	3.0	− 4.0312 from **10**	− H_4_ from **7**
237.1855	C_15_H_25_O_2_	237.1849	2.5	0.0000	Helminthosporol (**7**) [M+H]^+^
249.1488	C_15_H_21_O_3_	249.1485	1.2	− 4.0316 from **5**	− H_4_ from **5**
249.149	C_15_H_21_O_3_	249.1485	2.0	− 4.0314 from **5**	− H_4_ from **5**
251.165	C_15_H_23_O_3_	251.1642	3.2	− 2.0154 from **5**	− H_2_ from **5**
253.1804	C_15_H_25_O_3_	253.1798	2.4	15.9949 from **7**	**5** [M+H]^+^
263.2377	C_18_H_31_O	263.2369	3.0	42.047 from **6**	+ C_3_H_6_ from **6**
270.2072	C_15_H_28_NO_3_	270.2064	3.0	17.0268 from **5**	**5** [M+NH_4_]^+^
275.1627	C_15_H_24_O_3_Na	275.1623	1.5	37.9772 from **7**	Helminthosporic acid (**8**) [M+Na]^+^
287.1259	C_15_H_20_O_4_Na	287.1259	0.0	− 4.0312 from **1**	− H_4_ from **1**
289.1783	C_16_H_26_O_3_Na	289.178	1.0	14.0156 from **8**	+ CH_2_ from **8**
291.1571	C_15_H_24_O_4_Na	291.1572	0.3	15.9944 from **8**	**1** [M+Na]^+^
291.1574	C_15_H_24_O_4_Na	291.1572	0.7	15.9947 from **8**	**2** [M+Na]^+^
293.2483	C_19_H_33_O_2_	293.2475	2.7	56.0628 from **7**	+ C_4_H_8_ from **7**
301.1782	C_17_H_26_O_3_Na	301.178	0.7	26.0155 from **8**	**4** [M+Na]^+^
307.1911	C_18_H_27_O_4_	307.1904	2.3	− 2.0156 from **9**	− H_2_ from **9**
309.2067	C_18_H_29_O_4_	309.206	2.3	72.0212 from **7**	Sorokinianin (**9**) [M+H]^+^
309.2427	C_19_H_33_O_3_	309.2424	1.0	56.0623 from **5**	+ C_4_H_8_ from **5**
317.1732	C_17_H_26_O_4_Na	317.1729	0.9	42.0105 from **8**	**6** [M+Na]^+^
321.1682	C_18_H_25_O_5_	321.1697	4.7	11.9615 from **9**	**3** [M+H]^+^
333.1681	C_17_H_26_O_5_Na	333.1678	0.9	15.9949 from **6**	+ O from **6**
345.2045	C_19_H_30_O_4_Na	345.2042	0.9	28.0313 from **6**	+ C_2_H_4_ from **6**
375.2514	C_21_H_36_NO_4_	375.2511	0.8	84.0943 from **1**	+ C_6_H_12_ from **1**

^a^Δ = (Observed *m/z* − Calc. *m/z*)/Calc. *m/z*

### Structure elucidation of isolated sesquiterpenoids

**Fig. 3:**
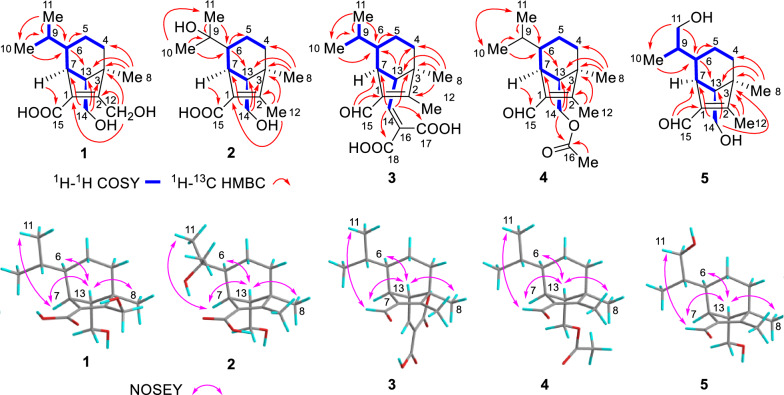
2D NMR correlations of compounds **1**–**5**. **A** Key ^1^H-^1^H COSY and ^1^H-^13^C HMBC correlations. **B** Key ROESY correlations

**Fig. 4:**
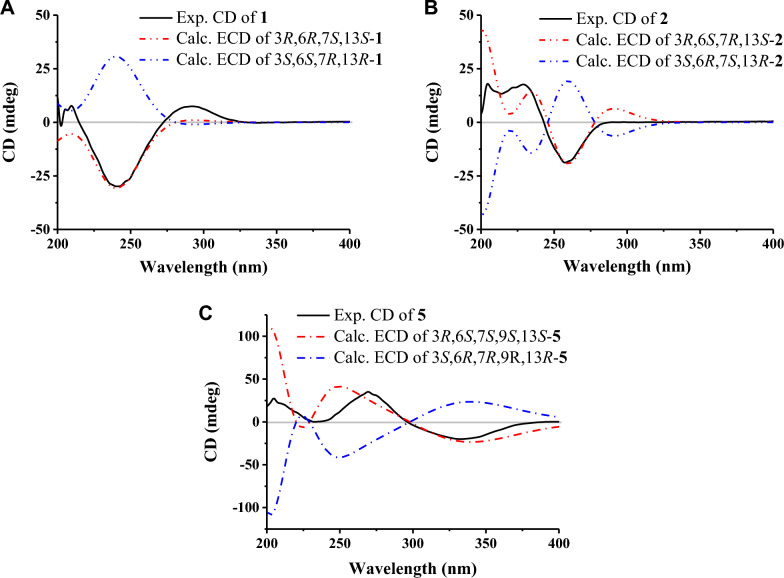
Experimental and computed ECD of compounds **1** (**A**), **2** (**B**), and **5** (**C**)

The molecular formula of compound **1** was C_15_H_24_O_4_ confirmed by its HRESIMS data ([M+Na]^+^ *m/z* 291.1567, calcd. for C_15_H_24_O_4_Na^+^ 291.1571) (Fig. S1a). Analysis of ^1^H NMR, ^13^C NMR, and HSQC spectra (Table 2 and Figs. S1b-S1 d) revealed a carboxyl cluster [*δ*_C_ 167.5], two olefinic carbons [*δ*_C_ 157.7, 128.5], four methylene (two of which were substituted with the hydroxyl groups [*δ*_C_ 55.6, 59.9]), four methine and three methyl groups, as well as one *sp*^3^ quaternary carbon, outlining a *seco*-sativene sesquiterpene skeleton. The ^1^H-^1^H COSY (Fig. S1e) revealed a continuous spin system extending from H_2_−4 to H_3_−10/H_2_−11. In addition, H-6 was further coupled with H-7, which also coupled with H-13, and H-13 coupled with H_2_−14 (Fig. 3 and Table S1). The two hydroxyl groups at C-12 (*δ*_C_ 55.6) and C-14 (*δ*_C_ 59.9) could be further determined by the HMBC (Fig. S1f) correlations of oxymethene protons at *δ*_H_ 4.54 (d, *J* = 13.2 Hz) and 4.12 (d, *J* = 13.2 Hz) with C-1 (*δ*_C_ 128.5), C-2 (*δ*_C_ 157.7) and C-3 (*δ*_C_ 49.2), and oxymethene protons at *δ*_H_ 3.44 (dd, *J* = 10.3, 4.9 Hz), and 3.05 (dd, *J* = 10.3, 9.4 Hz) with C-3, C-7 (*δ*_C_ 43.2) and C-13 (*δ*_C_ 62.0). A six-membered ring was formed on C-13 was connected to C-4 through C-3, according to the HMBC correlations between H_3_−8 and C-3, C-4, and C-13. The presence of a methyl group (Me-8) at C-3 was inferred as well. The olefinic carbons C-1 and C-2 were connected to C-7 and C-3, respectively, according to the HMBC correlations of H-7/C-15, H-7/C-1, H-7/C-2, H_2_−12/C-2, and H_2_−12/C-3. These observations suggested the structure of compound **1** closely resembled helminthosporic acid [27], with the notable substitution of a methyl group for a hydroxylmethylene group. The relative configurations of **1** could be established according to the ROESY (Fig. S1 g) correlations of H-7/H_3_−11, H-7/H-13, H_3_−8/H-13, and H-13/H-6, as well as comparison with literature [27] (Fig. 3). To figure out the absolute configurations of **1**, the electronic circle dichromatography (ECD) calculation of two epimers 3*R*,6*R*,7*S*,13*S*-**1**/3*S*,6*S*,7*R*,13*R*-**1** was performed, resulting in the 3*R*,6*R*,7*S*,13*S* configurations of **1** (Fig. 4A). Therefore, the structure of **1** was established (Fig. 2C) and it was named 12-hydroxyhelminthosporic acid.

**Table 2:** ^1^H and ^13^C NMR Data of compounds **1**–**5**

Pos	1^a^		2^b^		3^c^		4^c^		5^a^	
	*δ*_C_, mult	*δ*_H_, mult (*J* in Hz)	*δ*_C_, mult	*δ*_H_, mult (*J* in Hz)	*δ*_C_, mult	*δ*_H_, mult (*J* in Hz)	*δ*_C_, mult	*δ*_H_, mult (*J* in Hz)	*δ*_C_, mult	*δ*_H_, mult (*J* in Hz)
1	128.5, C		127.9, C		136.2, C		136.0, C		137.1, C	
2	157.7, C		156.1, C		165.7, C		166.0, C		168.7, C	
3	49.2, C		49.5, C		51.8, C		50.4, C		50.8, C	
4	34.9, CH_2_	1.48, m	33.1, CH_2_	1.33, m	32.6, CH_2_	1.50, m	33.1, CH_2_	1.39, m	34.3, CH_2_	1.43, m
		1.26, m		1.28, m		1.30, m				
5a	24.8, CH_2_	1.64, m	20.4, CH_2_	1.59, m	24.6, CH_2_	1.70, m	24.7, CH_2_	1.66, m	25.4, CH_2_	1.73, m
5b		1.09, m		1.28, m		0.85, m		0.81, m		0.90, m
6	44.8, CH	0.97, m	48.4, CH	1.30, m	43.7, CH	1.00, m	44.1, CH	0.98, m	40.3, CH	1.43, m
7	43.2, CH	3.09, brs	41.7, CH	3.06, brs	44.4, CH	2.83, brs	41.0, CH	2.94, brs	41.4, CH	3.39, brs
8	18.7, CH_3_	0.99, s	18.9, CH_3_	0.88, s	18.9, CH_3_	0.93, s	18.0, CH_3_	1.00, s	18.4, CH_3_	1.02, s
9	31.4, CH	1.16, m	70.5, C		31.0, CH	0.94, m	31.1, CH	0.94, m	39.2, CH	1.11, m
10	21.8, CH_3_	0.97, d (6.3)	28.5, CH_3_	1.04, s	21.5, CH_3_	0.97, d (6.0)	21.5, CH_3_	0.99, d (6.3)	15.8, CH_3_	0.80, d (6.9)
11	20.8, CH_3_	0.76, d (6.3)	28.0, CH_3_	1.13, s	20.5, CH_3_	0.73, d (6.0)	20.5, CH_3_	0.73, d (6.5)	67.7, CH_2_	3.87, dd (11.1, 3.9)
										3.62, dd (11.1, 4.7)
12	55.6, CH_2_	4.54, d (13.2)	12.2, CH_3_	1.88, s	10.5, CH_3_	2.08, s	10.3, CH_3_	2.03, s	10.9, CH_3_	2.03, s
		4.12, d (13.2)								
13	62.0, CH	1.42, dd (9.4, 4.9)	63.3, CH	1.50, dd (9.5, 5.2)	59.6, CH	2.42, d (10.9)	56.9, CH	1.75, dd (9.0, 5.5)	61.0, CH	1.73, dd (9.9. 4.7)
14	59.9, CH_2_	3.44, dd (10.3, 4.9)	59.8, CH_2_	3.40, dd (10.2, 5.2)	145.6, CH	6.51, d (10.9)	63.6, CH_2_	4.00, dd (11.1, 5.5)	61.8, CH_2_	3.62, dd (11.1, 4.7)
		3.05, dd (10.3, 9.4)		3.06, dd (10.2, 9.5)				3.66, dd (11.1, 8.9)		3.26, dd (11.1, 9.9)
15	167.5, C		168.7, C		188.6, CH	10.03, s	188.4, CH	9.99, s	189.4, CH	10.00, s
16					131.2, C		170.4, C			
17					164.9, C		20.7, CH_3_	1.98, s		
18					166.8, C					

^a^Recorded in CDCl_3_, ^1^H 500 MHz; ^13^C 125 MHz; ^b^Recorded in DMSO-*d*_6_, ^1^H 500 MHz; ^13^C 125 MHz; ^c^Recorded in DMSO-*d*_6_, ^1^H 800 MHz; ^13^C 200 MHz

Compound **2** has a molecular formula of C_15_H_24_O_4_, as confirmed by HRESIMS data (Fig. S2a). Comparative analysis of ^1^H and ^13^C NMR data (Table 2 and Fig. S2b–d) between compounds **1** and **2** revealed that two secondary methyl groups [*δ*_C_/*δ*_H_ 20.8/0.76 (d, *J* = 6.3 Hz), *δ*_C_/*δ*_H_ 21.8/0.97 (d, *J* = 6.3 Hz)] were replaced by two methyl groups at lower field [*δ*_C_/*δ*_H_ 28.0/1.13 (s), *δ*_C_/*δ*_H_ 28.5/1.04 (s)]. The appearance of an oxygenated quaternary carbon (*δ*_C_ 70.5) suggested the presence of one hydroxyl group at C-9. HMBC (Fig. 3 and Supplementary Fig. S2e, f) further confirmed this hypothesis with correlations of H_3_−10/C-6, H_3_−10/C-9, H_3_−10/C-11. Resonances at *δ*_H_ 1.88 (s) showed correlations with carbons at *δ*_C_ 49.5(C-3), *δ*_C_ 127.6(C-1), and *δ*_C_ 156.1(C-2), establishing the methyl group location of C-12. The relative configurations of **2** were consistent with those of **1** by detailed analysis of their ROESY (correlations of H-7/H_3_−11, H-7/H-13, H_3_−8/H-13, and H-13/H-6) (Fig. 3, Fig. S2 g, and Table S2). The absolute configurations were assigned as 3*R*,6*S*,7*R*,13*S* through ECD calculation. Thus, the structure of **2** was established (Fig. 2C), and this compound was named 9-hydroxyhelminthosporic acid.

The molecular formula of compound **3** was deduced as C_18_H_24_O_5_ based on HRESIMS (Fig. S3a) (*m/z* 343.1514 [M+Na]^+^, calcd. for 343.1516) with 7 degrees of unsaturation. ^1^H, ^13^C and 2D NMR data (Figs. S3b–f) revealed a similar structural skeleton with compound **1**, except for resonances of two additional carboxyls (*δ*_C_ 164.9, 166.8) and one olefinic bond (*δ*_C_ 145.6, 131.2). HMBC correlations from H-14 (*δ*_H_ 6.51 d, *J* = 10.9 Hz) to C-3, C-7, C-16, C17, and C-18 revealed that two carboxyl groups were connected with C-14 through non-protonated carbon C-16. The relative configurations of **3** were consistent with **1** based on key ROESY correlations of H-7/H_3_−11, H-7/H-13, H_3_−8/H-13, and H-13/H-6 (Fig. 3, Fig. S3 g, and Table S3). Given the shared biosynthetic pathway of compounds **1** and **3** through the *seco*-sativene sesquiterpene skeleton, the absolute configurations of **3** were deduced to align with that of **1**, and was designated 3*R*,6*R*,7*S*,13*S*. This alignment was supported by its specific optical rotation ([α]−20) in contrast to compound **1** ([α]−38) and to bipolarisorokin H ([α]−137) [28]. Thus, **3** was a new compound (Fig. 2C) and named bipolarisorokin I.

HRESIMS of **4** displayed a molecular ion peak at *m/z* 301.1775 for [M+Na]^+^ (calcd. for 301.1774) (Fig. S4a) and indicating a molecular formula of C_17_H_26_O_3_. 1D and 2D NMR data (Figs. S4b–f) showed that compound **4** shared a structural framework with helminthosporol (**7**) [29], distinguished by an additional acetyl group [*δ*_C_/*δ*_H_ 20.7/1.98 (s), *δ*_C_ 170.4]. HMBC correlations from H_3_−17 and H_2_−14 to C-16 confirmed compound **4** as a 14-OH acetylated analogue of **7**. Through ROESY analysis, the crosspeaks of H-7/H_3_−11, H-7/H-13, H_3_−8/H-13, and H-13/H-6 revealed that the relative configurations of **4** were consistent with those of **1** (Fig. 3, Fig. S4 g, and Table S4). Its absolute configurations were deduced as 3*R*,6*R*,7*S*,13*S* considering its biosynthetic lineage from *seco*-sativene sesquiterpene, and comparison of specific rotation values (**4**, [α]+11.0) with literature (bipolenin H, [α]+17.5) [30]. Accordingly, compound **4** was definitively identified and named bipolarisorokin J, with a defined structure (Fig. 2C). The NMR data of compound **4** is reported here for the first time.

**Fig. 5:**
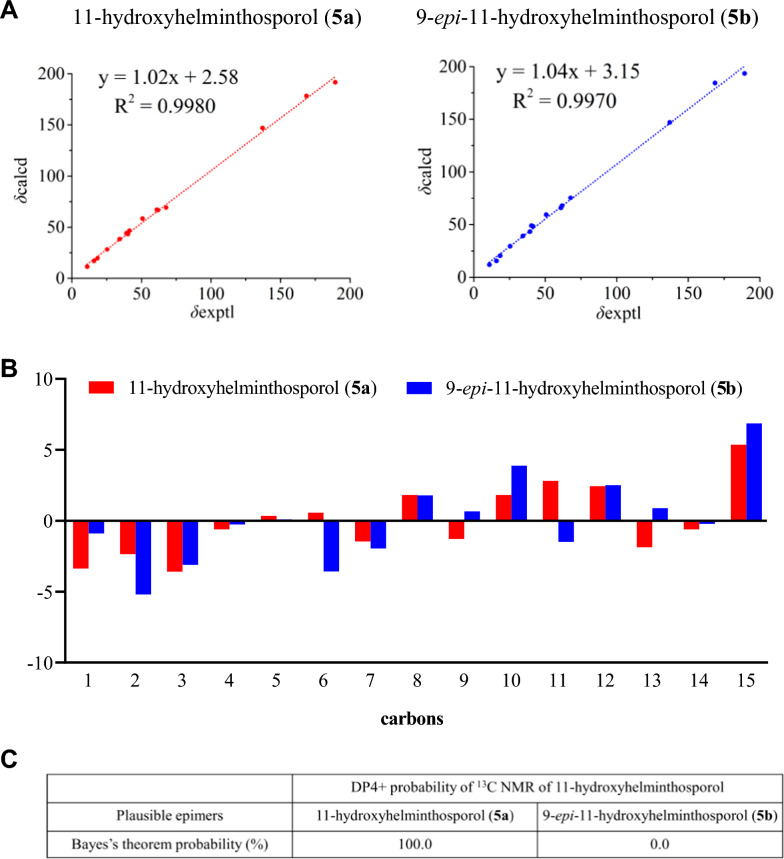
^13^C NMR calculation results of two plausible isomers **5a**/**5b** at the B3LYP/6–311++G(2 d,p) level. **A** Linear correlation plots of the calculated and experimental ^13^C NMR values. **B** Relative errors between the calculated and recorded ^13^C values. **C** DP4+ probability

Compound **5** possessed a molecular formula of C_15_H_24_O_3_ (four degrees of unsaturation) determined by HRESIMS data (Fig S5a). NMR analysis (^1^H and ^13^C, Table 2 and Figs. S5b-S5 d) revealed a structural resemblance to helminthosporol (**7**), except that the methyl group (Me-11) was replaced by a hydroxylmethylene group. The presence of a hydroxyl group at C-11 could be assigned by HMBC (Fig. 3 and Fig. S5e, f) correlations of the downfield shifted signals at *δ*_H_ 3.87 (dd, *J* = 11.1, 3.9 Hz) and 3.62 (dd, *J* = 11.1, 4.7 Hz) with C-6, C-9 and C-10. Detailed analysis of ROESY afforded the 3R*,6S*,7S*,13S* relative configuration in **5** (Fig. 4B, Fig. S5 g, Table S5). The relative configurations between C-6 and C-9 in **5** was determined to be 6S*,9S* by ^13^C NMR calculations and DP4+ analyses (Fig. 5, Table S9) [31]. ECD calculations confirmed the absolute configurations of **5** as 3*R*,6*S*,7*S*,9*S*,13*S*, leading to the identification of compound **5** as 11-hydroxyhelminthosporol (Fig. 2) [32].

Compounds **6**–**10** were identified as helminthosporic acid derivative (**6**) [33], helminthosporol (**7**) [29], helminthosporic acid (**8**) [27], sorokinianin (**9**) [34], and secolongifolene diol (**10**) [29], by comparing their spectroscopic data with those in previous literature.

### Proposed biosynthetic pathway of the isolated sesquiterpenoids

**Fig. 6:**
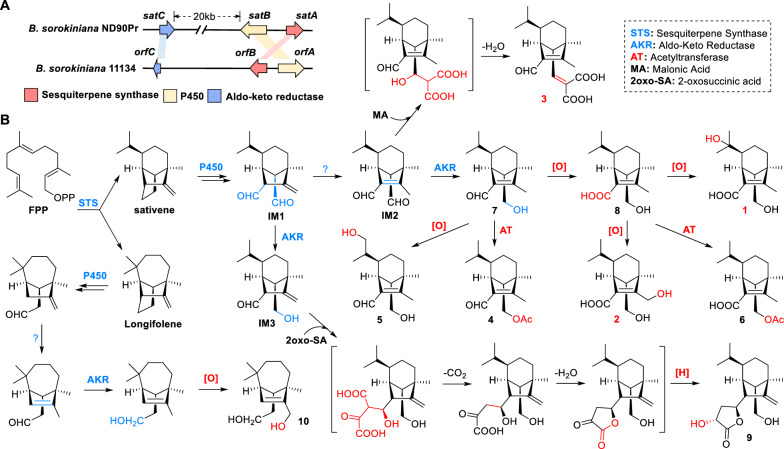
Organization of the *seco*-sativene biosynthetic gene cluster in *B. sorokiniana* ND90Pr and BS11134 (**A**), and proposed biosynthetic pathway for isolated sesquiterpenoids **1**–**10** (**B**)

Sativene could be synthesized from FPP by sesquiterpene synthase (Fig. 6), by a mechanism involving ionization and successive rearrangement [35]. Compounds **1**–**9** belong to *seco*-sativene type irregular terpenoids, while compound **10** belongs to *seco*-longifolene type sesquiterpenoids [29]. The plausible biosynthetic routes of compounds **1**–**10** were described based on recent research [26, 36] (Fig. 6).

Intermediate **IM2** was generated from sativene through successive oxidation by P450 and a spontaneous shift of the *Δ*^2,12^ olefinic bond. Subsequent reduction of the C-14 aldehyde, catalysed by a Aldo–keto reductase (AKR) yielded compound **7**. Compounds **4**, **5**, and** 8** can be derived from Compound **7** through acetylation at 14-OH, hydroxylation at C-11, and carboxylation of C-15, respectively. Hydroxylation at C-9 or C-12 of compound **8** yielded compounds **1** and **2**, respectively*.* Compound **6** resulted from the acetylation at 14-OH of Compound **8**. The γ-butyrolactone moiety in Compound **9** was proposed to derive from the TCA cycle intermediate 2-oxosuccinic acid via an aldol condensation reaction, as supported by previous isotope labelling studies [37]. A similar process occurred with compound **3**, where the 2-methylenemalonic acid moiety was incorporated using malonic acid as intermediate through an aldol condensation reaction with C-14 aldehyde of **IM2** (Fig. 6B). *seco*-Longifolene type sesquiterpenoid **10** was postulated to possess a divergent cyclization route, generating an end product with a seven-membered ring [27, 38].

### In vitro anti-inflammatory assay

All isolated sesquiterpenoids from strain BS11134 were screened for their anti-inflammatory activity (Table 3) by evaluating inhibition effects on the NO production induced by LPS in RAW264.7, a mouse macrophage cell line. Compounds **1** and **9** exhibited anti-inflammatory effects in vitro, with inflammation inhibition rates of 28.0 ± 2.4% and 84.7 ± 1.7%, respectively, at a concentration of 10 μM, compared to the positive control, indomethacin, which showed a 51.2 ± 8.2% inhibition rate.

**Table 3:** NO inhibitory activities of compounds

Compound	% NO inhibition (10 μM)
**1**	28.0 ± 2.4
**2**	10.9 ± 2.3
**8**	14.4 ± 2.5
**9**	84.7 ± 1.7
Indomethacin	51.2 ± 2.0

## Discussion and conclusion

The fungal kingdom is a primary source of new natural products, yet the capabilities of only a few species have been uncovered [39]. Terpenoids represent a crucial class of natural products derived from filamentous fungi. Fungal sesquiterpenoids, in particular, are significant targets for medical drug design because of their potential bioactivity [14, 40]. Our research uncovered ten sesquiterpenoids with a sativene architecture from the phytopathogenic fungus *B. sorokiniana* 11134.

Several structural classes related to our isolated compounds have been previously reported from related members of the family Pleosporaceae including species of *Bipolaris*, *Cochliobolus*, and *Drechslera*; such compounds include sativene, *seco*-sativene, isosativene, longifolene, and *seco*-longifolene. We expand the reported *seco*-sativene repertoire with three new congeners **1**–**3**. *seco*-sativenes sesquiterpenoids, characterized by a bicyclo[3.2.1]octane structure (6–5 ring system) [41], are biosynthetically generated from sativene through the C14–C15 bond cleavage, and undergo various post-modifications such as isomerization, ketone reduction, acetylation, carboxylation, and glycosylation [41].

Recently, Zhang et al*.* revealed the biosynthetic gene cluster of *seco*-sativenes in *B. sorokiniana* ND90Pr through heterologous expression of a three-gene cassette in *Aspergillus nidulan*s. The terpene cyclase (SatA) responsible for forming the 6-5-5 ring sativene skeleton, the cytochrome P450 (SatB) involved in C14–C15 bond cleavage, and the aldo–keto reductase (SatC) for the regioselective reduction of C14 aldehyde have been characterized [26]. We detected a similar three-gene cassette in strain BS11134, responsible for the biosynthesis of compounds **1**–**10,** showing high similarity to the *sat* cluster (Fig. 6A). The proposed biosynthetic pathway for compounds **1**–**10** suggested that additional, yet unidentified enzymes (acetylase, oxidoreductase, double-bond translocase, etc.) are required for transforming the key intermediate **IM1** into these sesquiterpenoids (Fig. 6B).

Currently*,* more than 40 *seco*-sativenes featuring the bicyclo[3.2.1]octane structure have been isolated from fungi, exhibiting diverse bioactivities such as phytotoxicity, growth promotion, anti-NO production, ACAT inhibition, and antifungal effects [41]. In this study, we reported the NO production inhibition activity of sorokinianin (84.7 ± 1.7% at 10 μM) for the first time, which has been recognized as phytotoxin for decades [34]. Previously, only a few *seco*-sativene sesquiterpenoids were known for their anti-inflammatory potential. For instance, bipolenins G and 9-hydroxyhelminthosporol demonstrated anti-NO production activities with IC_50_ values of 23.8 and 17.5 μM, respectively [30].

In summary, feature-based molecular networking generated from GNPS confirmed the cluster of sesquiterpenoids, and HRMS-oriented isolation led to the characterization of ten sesquiterpenoids with a helminthoporene skeleton, including three new compounds (**1**–**3**), alongside the first detailed NMR data for compound 4. The structures of these compounds were unequivocally determined through comprehensive analyses of MS and NMR data. This research extends the known helminthoporene class of molecules and underscores the untapped potential of phytopathogenic fungi as sources of novel compounds.

## Materials and methods

### General experimental section

NMR spectra were recorded using a Bruker Advance DRX600 spectrometer and Bruker Advance III HDX 800 MHz spectrometer. Deuterated solvents (CDCl_3_ and DMSO-*d*_6_) were purchased from Cambridge Isotope Laboratories (CIL). A Bruker Maxis II ETD QTOF mass spectrometer was used to run HRESIMS analysis. Column chromatography was performed with Sephadex LH-20 (GE Healthcare BioSciences AB) and ODS-A (YMC, Japan). Semi-preparative HPLC was carried out equipped with Phenomenex Luna C18 (5 μm, 9.4 × 250 mm), Eclipse XDB-C3 (5 μm, 9.4 × 250 mm), and ZORBAX RX-C8 (9.4 × 250 mm) columns. HPLC analysis were carried out with an Agilent 1100 Series separation module coupled with DAD detector. The UV–vis spectra were measured using a Cary 50 spectrophotometer. The optical rotation was recorded on a Perkin-Elmer Model 343 polarimeter. Biological media, reagents, and chemicals were obtained from standard commercial sources.

### Characterization and identification of pathogenic fungus BS11134

The original culture of BS11134 was obtained from a leaf of *Poa pratensis* collected from Sujiatun (GPS 40.21432, 116.53574), Chaoyang District, Beijing, in July 2013. It was characterized as *Bipolaris sorokiniana* by ITS DNA gene sequence (accession no. KU297882) and morphology [42], and has been deposited in the China General Microbiological Culture Collection Center (accession No. 3.18317), which is a member of the World Data Centre for Microorganisms (WDCM 550).

### GNPS directed dereplication and prediction of new sesquiterpenoids

Samples were dissolved in MeOH to make 1 mg/mL solution and eluted with a gradient of H_2_O (1‰ HCOOH) and CH_3_CN with a gradient method as follows: 5% CH_3_CN gradient elution to 99% in 9 min, 99% CH_3_CN kept for 3 min, 99% CH_3_CN changed to 5% in 0.1 min, and 5% CH_3_CN kept for 3 min with the flow rate of 0.35 mL/min. LC–MS/MS data was acquired on Bruker Maxis II ETD QTOF mass spectrometer coupled with Thermo scientific UltiMate 3000 HPLC system. The raw data was converted to the mzXML file format using the Bruker Data Analysis software. The.mzML file was processed using the MZmine (version 2.53). The mass detections were realized keeping the noise level at 1.0E^3^ for MS1 and 1.0E^1^ for MS2, respectively. The ADAP chromatogram building used a minimum time span of 0.1 min, *m*/*z* tolerance of 0.02 (or 5 ppm), and minimum height of 1.0E^3^. Deisotop was carried out utilizing isotopic peaks grouper algorithm, with a RT tolerance of 0.1 min and *m*/*z* tolerance of 0.001 (or 5 ppm). Duplicate peaks were filtered with filter mode of old average, *m*/*z* tolerance of 0.001 (or 5 ppm), and retention time (RT) range = 1 min. A feature-based molecular networking was created using the web-based workflow (version release_28.2) at GNPS (https://gnps.ucsd.edu/) [17, 19]. Cytoscape (version 3.7.2) was used to visualize the corresponding output FBMN data.

### Scale-up fermentation and secondary metabolites purification

The strain BS11134 was cultured on potato dextrose agar (PDA) at 28 °C for 10 days. The well-grown agar cultures were cut into small cubes (0.5 × 0.5 × 0.5 cm^3^) and used to inoculate 250 mL Erlenmeyer flasks containing 50 mL PDB medium, which were further incubated on a rotary shaker at 28 °C, 170 rpm for 5 days to generate the seed culture. The scale-up fermentation was carried out with twenty 1000 mL Erlenmeyer flasks, each containing 160 g of rice and 240 mL of distilled water, soaked overnight, followed by being autoclaved at 15 psi for 30 min. Then each flask was inoculated with 12 mL of the seed culture, and grown at 28 °C for 40 days.

The fermentation products were extracted exhaustively with EtOAc and were concentrated in vacuo. The crude extracts (27.7 g) were separated by silica gel, Sephadex LH-20 column chromatography and ODS-MPLC [43], to obtain sub-fractions containing the sesquiterpenoids, guided by the molecular networking and LC–MS results. Among these sub-fractions, E1 C7D (89 mg) was further separated by preparative-HPLC running with Eclipse XDB-C8 (9.4 × 250 mm) column at a flow rate of 3.0 mL/min eluting with the following gradient: 0 min, 50% CH_3_CN-H_2_O; 30 min 50% CH_3_CN; 60 min 70% CH_3_CN to obtain **7** (1.5 mg, *t*_R_ = 13.9 min). E6B was seperated by ODS-MPLC using gradient elution from 50 to 100% CH_3_OH-H_2_O for 170 min to obtain eleven fractions (E6B1–E6B11). E6B9 was purified on a Sephadex LH-20 column using CH_3_OH to obtain compound **4** (1.0 mg). E6B7-2 (100 mg) was purified by preparative RP-HPLC using an Eclipse XDB-C8 (9.4 × 250 mm) column eluting at a flow rate of 3.0 mL/min using a gradient elution: 0 min, 20% CH_3_CN–H_2_O; 30 min 20% CH_3_CN–H_2_O; 42 min 23% CH_3_CN–H_2_O to obtain **1** (9.5 mg, *t*_R_ = 40.1 min). E6B7–10 (145 mg) was purified by preparative RP-HPLC using an ZORBAX RX-C8 (9.4 × 250 mm) column eluting at a flow rate of 3.0 mL/min using a gradient elution: 0 min, 25% CH_3_CN–H_2_O; 26 min 34% CH_3_CN-H_2_O to obtain **2** (22.0 mg, *t*_R_ = 21.4 min). E6B7–8 (142 mg) was purified by preparative RP-HPLC using an ZORBAX RX-C8 (9.4 × 250 mm) column eluting at a flow rate of 3.0 mL/min using a gradient elution: 0 min, 20% CH_3_CN–H_2_O; 24 min 36% CH_3_CN–H_2_O to obtain **3** (26.2 mg, *t*_R_ = 16.6 min). E2 C6 (470 mg) was purified by preparative RP-HPLC using an Phenomenex Luna C18 column (5 μm, 9.4 × 250 mm) eluting at a flow rate of 4.0 mL/min using a gradient elution: 0 min, 60% CH_3_OH–H_2_O; 25 min 95% CH_3_OH–H_2_O to obtain **5** (1.0 mg, *t*_R_ = 18.3 min). E2D3 (349 mg) was purified by preparative RP-HPLC using an Eclipse XDB-C3 column (5 μm, 9.4 × 250 mm) eluting at a flow rate of 3.0 mL/min using a gradient elution: 0 min, 40% CH_3_CN–H_2_O; 25 min 60% CH_3_OH–H_2_O to obtain **6** (2.1 mg, *t*_R_ = 21.3 min). Fraction E3 was fractionated on a Sephadex LH-20 column using CH_2_Cl_2_–CH_3_OH (1:1) to give 33 fractions (1–33). Sub-fraction E3–24 (150 mg) was purified by preparative RP-HPLC using an ZORBAX RX-C8 (9.4 × 250 mm) column eluting at a flow rate of 3.0 mL/min using a gradient elution: 0 min, 35% CH_3_CN–H_2_O; 50 min 45% CH_3_CN–H_2_O to obtain **8** (9.9 mg, *t*_R_ = 30.1 min) and **9** (4.4 mg, *t*_R_ = 40.0 min). E3D4 (190 mg) was purified by preparative RP-HPLC using an Phenomenex Luna C18 column (5 μm, 9.4 × 250 mm) eluting at a flow rate of 4.0 mL/min using a gradient elution: 0 min, 50% CH_3_OH–H_2_O; 25 min 80% CH_3_OH-H_2_O to obtain **10** (2.2 mg, *t*_R_ = 20.0 min).

### Quantum chemical computation details of ECD and ^13^C NMR spectra

Calculations were performed using the density functional theory (DFT) as carried out in Gaussian 03 [44], with the methods described in previous study [42, 45].

### In vitro anti-inflammatory assay

The in vitro anti-inflammatory assay followed the methods of Yang et al. [46].

## Supplementary Information

Supplementary Material 1

## Data Availability

Data will be made available on request.
