# Supplementary material for: New sesquiterpenoids with anti-inflammatory effects from phytopathogenic fungus Bipolaris sorokiniana 11134
Source: Nat Prod Bioprospect. 2025 May 9;15(1):29. doi: 10.1007/s13659-025-00508-9 (PMC12064533; doi:10.1007/s13659-025-00508-9)
Supplement: Supplementary file 1 — Supplementary Material 1 [file 13659_2025_508_MOESM1_ESM.docx]

**Supporting Information**

**New sesquiterpenoids with anti-inflammatory effects from phytopathogenic fungus *Bipolaris sorokiniana* 11134**

Qiang Yin^a,†^, Jianying Han^a,b,c†^, Guixiang Yang^a^, Zhijun Song^d^, Keke Zou^a^, Kangjie Lv^a^, Zexu Lin^a^, Lei Ma^a^, Miaomiao Liu^b^, Yunjiang Feng^b^, Ronald J Quinn^b^, Tom Hsiang^e^, Lixin Zhang^a^, Xueting Liu^a^, Guoliang Zhu^a,^*, Jingyu Zhang^a,^*

**^a^**State Key Laboratory of Bioreactor Engineering, East China University of Science and Technology, Shanghai 200237, China

**^b^**Griffith Institute for Drug Discovery, Griffith University, Brisbane, QLD, Australia

^c^Institute for Molecular Bioscience, The University of Queensland, St. Lucia, QLD 4072, Australia

^d^Chinese Academy of Sciences, Key Laboratory of Pathogenic Microbiology and Immunology, Institute of Microbiology, Chinese Academy of Sciences, Beijing, China

^e^School of Environmental Sciences, University of Guelph, Guelph, Ontario N1G 2W1, Canada

^†^The authors contributed to this work equally.

*Corresponding author: Guoliang Zhu, Jingyu Zhang

E-mail: [zhuguoliang@ecust.edu.cn](mailto:zhuguoliang@ecust.edu.cn) (G. Zhu); [zhangjingyu@ecust.edu.cn](mailto:zhangjingyu@ecust.edu.cn) (J. Zhang)

Phone (Fax): CN +86-021-64253020.

**Table of contents**

[**Fig. S1a** HRESIMS spectrum of compound **1** in MeOH 5](#_Toc176855066)

[**Fig. S1b** ^1^H NMR (500 MHz, DMSO-*d_6_*) spectra of compound **1** 5](#_Toc176855067)

[**Fig. S1c** ^13^C NMR (125 MHz, DMSO-*d_6_*) spectrum of compound **1** 6](#_Toc176855068)

[**Fig. S1d** HSQC (500 MHz, DMSO-*d_6_*) spectrum of compound **1** 6](#_Toc176855069)

[**Fig. S1e** ^1^H-^1^H COSY (500 MHz, DMSO-*d_6_*) spectrum of compound **1** 7](#_Toc176855070)

[**Fig. S1f** HMBC (500 MHz, DMSO-*d_6_*) spectrum of compound **1** 7](#_Toc176855071)

[**Fig. S1g** ROESY (500 MHz, DMSO-*d_6_*) spectrum of compound **1** 8](#_Toc176855072)

[**Table S1** NMR data of compound **1** 8](#_Toc176855073)

[**Fig. S2a** HRESIMS spectrum of compound **2** in MeOH 9](#_Toc176855074)

[**Fig. S2b** ^1^H NMR (500 MHz, DMSO-*d_6_*) spectra of compound **2** 9](#_Toc176855075)

[**Fig. S2c** ^13^C NMR (125 MHz, DMSO-*d_6_*) spectrum of compound **2** 10](#_Toc176855076)

[**Fig. S2d** HSQC (500 MHz, DMSO-*d_6_*) spectrum of compound **2** 10](#_Toc176855077)

[**Fig. S2e** ^1^H-^1^H COSY (500 MHz, DMSO-*d_6_*) spectrum of compound **2** 11](#_Toc176855078)

[**Fig. S2f** HMBC (500 MHz, DMSO-*d_6_*) spectrum of compound **2** 11](#_Toc176855079)

[**Fig. S2g** ROESY (500 MHz, DMSO-*d_6_*) spectrum of compound **2** 12](#_Toc176855080)

[**Table S2** NMR data of compound **2** 12](#_Toc176855081)

[**Fig. S3a** HRESIMS spectrum of compound **3** in MeOH 13](#_Toc176855082)

[**Fig. S3b** ^1^H NMR (800 MHz, DMSO-*d*_6_) spectra of compound **3** 13](#_Toc176855083)

[**Fig. S3c** ^13^C NMR (200 MHz, DMSO-*d*_6_) spectrum of compound **3** 14](#_Toc176855084)

[**Fig.** **S3d** HSQC (800 MHz, DMSO-*d*_6_) spectrum of compound **3** 14](#_Toc176855085)

[**Fig. S3e** ^1^H-^1^H COSY (800 MHz, DMSO-*d*_6_) spectrum of compound **3** 15](#_Toc176855086)

[**Fig.** **S3f** HMBC (800 MHz, DMSO-*d*_6_) spectrum of compound **3** 15](#_Toc176855087)

[**Fig. S3g** ROESY (800 MHz, DMSO-*d*_6_) spectrum of compound **3** 16](#_Toc176855088)

[**Table S3** NMR data of compound **3** 16](#_Toc176855089)

[**Fig. S4a** HRESIMS spectrum of compound **4** in MeOH 18](#_Toc176855090)

[**Fig. S4b** ^1^H NMR (800 MHz, DMSO-*d*_6_) spectra of compound **4** 18](#_Toc176855091)

[**Fig. S4c** ^13^C NMR (200 MHz, DMSO-*d*_6_) spectrum of compound 4 19](#_Toc176855092)

[**Fig.** **S4d** HSQC (800 MHz, DMSO-*d*_6_) spectrum of compound **4** 20](#_Toc176855093)

[**Fig.** **S4f** HMBC (800 MHz, DMSO-*d*_6_) spectrum of compound **4** 21](#_Toc176855094)

[**Fig. S4g** ROESY (800 MHz, DMSO-*d*_6_) spectrum of compound **4** 21](#_Toc176855095)

[**Table S4** NMR data of compound **4** 21](#_Toc176855096)

[**Fig. S5a** HRESIMS spectrum of compound **5** in MeOH 23](#_Toc176855097)

[**Fig. S5b** ^1^H NMR (500 MHz, CDCl3) spectra of compound **5** 23](#_Toc176855098)

[**Fig. S5c** ^13^C NMR (125 MHz, CDCl3) spectrum of compound **5** 24](#_Toc176855099)

[**Fig. S5d** HSQC (500 MHz, CDCl3) spectrum of compound **5** 24](#_Toc176855100)

[**Fig. S5e** ^1^H-^1^H COSY (500 MHz, CDCl3) spectrum of compound **5** 25](#_Toc176855101)

[**Fig. S5f** HMBC (500 MHz, CDCl3) spectrum of compound **5** 25](#_Toc176855102)

[**Fig. S5g** ROESY (500 MHz, CDCl3) spectrum of compound **5** 26](#_Toc176855103)

[**Table S5** NMR data of compound **5** 26](#_Toc176855104)

[**Fig. S6a** HRESIMS spectrum of compound **6** in MeOH 27](#_Toc176855105)

[**Fig. S6b** ^1^H NMR (800 MHz, DMSO-*d*_6_) spectra of compound **6** 27](#_Toc176855106)

[**Fig. S6c** ^13^C NMR (200 MHz, DMSO-*d*_6_) spectrum of compound **6** 28](#_Toc176855107)

[**Fig. S6d** HSQC (800 MHz, DMSO-*d*_6_) spectrum of compound **6** 29](#_Toc176855108)

[**Fig. S6e** ^1^H-^1^H COSY (800 MHz, DMSO-*d*_6_) spectrum of compound **6** 30](#_Toc176855109)

[**Fig. S6f** HMBC (800 MHz, DMSO-*d*_6_) spectrum of compound **6** 31](#_Toc176855110)

[**Fig. S6g** ROESY (800 MHz, DMSO-*d*_6_) spectrum of compound **6** 32](#_Toc176855111)

[**Table S6** NMR data of compound **6** 32](#_Toc176855112)

[**Fig. S7a** HRESIMS spectrum of compound **7** in MeOH 34](#_Toc176855113)

[**Fig. S7b** ^1^H NMR (500 MHz, CDCl3) spectra of **7** 34](#_Toc176855114)

[**Fig. S7c** ^13^C NMR (125 MHz, CDCl3) spectrum of **7** 35](#_Toc176855115)

[**Fig. S8a** HRESIMS spectrum of compound **8** in MeOH 35](#_Toc176855116)

[**Fig. S8b** ^1^H NMR (500 MHz, CDCl3) spectra of **8** 36](#_Toc176855117)

[**Fig. S8c** ^13^C NMR (125 MHz, CDCl3) spectrum of **8** 36](#_Toc176855118)

[**Fig. S9a** HRESIMS spectrum of compound **9** in MeOH 37](#_Toc176855119)

[**Fig. S9b** ^1^H NMR (500 MHz, DMSO-*d*_6_) spectra of **9** 37](#_Toc176855120)

[**Fig. S9c** ^13^C NMR (125 MHz, DMSO-*d*_6_) spectrum of **9** 38](#_Toc176855121)

[**Table S7** ^1^H and ^13^C NMR Data of compounds **7**–**9** 38](#_Toc176855122)

[**Fig. S10a** HRESIMS spectrum of compound **10** in MeOH 39](#_Toc176855123)

[**Fig. S10b** ^1^H NMR (800 MHz, DMSO-*d*_6_) spectra of compound **10** 39](#_Toc176855124)

[**Fig. S10c** ^13^C NMR (200 MHz, DMSO-*d*_6_) spectrum of compound **10** 40](#_Toc176855125)

[**Fig. S10d** HSQC (800 MHz, DMSO-*d*_6_) spectrum of compound **10** 40](#_Toc176855126)

[**Fig. S10e** HMBC (800 MHz, DMSO-*d*_6_) spectrum of compound **10** 41](#_Toc176855127)

[**Table S8** NMR data of compound **10** 41](#_Toc176855128)

[**Table S9.** DP4 probability of ^13^C NMR chemical shifts of **5a** (3*R**,6*S**,7*S**,9*S**,13*S**) and **5b** (3*R**,6*S**,7*S**,9*R**,13*S**). 42](#_Toc176855129)

Compound **1**: colorless oil; [*α*]25 D -37.99 (*c* 0.10, MeOH); UV (MeOH) *λ*_max_ nm (log *ε*) 204 (3.34), 247 (3.52); ^1^H and ^13^C NMR data see Table 2; ^1^H-^1^H COSY and HMBC data see Supplementary Table S1; HRESIMS *m/z* 291.1567 (calcd for C_15_H_24_O_4_Na, 291.1567).

Compound **2**: colorless oil; [*α*]25 D -45.99 (*c* 0.10, MeOH); UV (MeOH) *λ*_max_ nm (log *ε*) 203 (3.30), 246 (3.58); ^1^H and ^13^C NMR data see Table 2; ^1^H-^1^H COSY and HMBC data see Supplementary Table S2; HRESIMS *m/z* 291.1567 (calcd for C_15_H_24_O_4_Na, 291.1567).

Compound **3**: colorless oil; [*α*]25 D -20 (*c* 0.10, MeOH); UV (MeOH) *λ*_max_ nm (log *ε*) 216 (3.83), 264 (3.68); ^1^H and ^13^C NMR data see Table 2; ^1^H-^1^H COSY and HMBC data see Supplementary Table S3; HRESIMS *m/z* 343.1514 (calcd for C_18_H_24_O_5_Na, 343.1516).

Compound **4**: colorless oil; [*α*]25 D +11 (*c* 0.06, MeOH); UV (MeOH) *λ*_max_ nm (log *ε*) 203 (3.66), 243 (3.57); ^1^H and ^13^C NMR data see Table 2; ^1^H-^1^H COSY and HMBC data see Supplementary Table S4; HRESIMS *m/z* 301.1775 (calcd for C_17_H_26_O_3_Na, 301.1774).

Compound **5**: colorless oil; [*α*]25 D -12 (*c* 0.10, MeOH); UV (MeOH) *λ*_max_ nm (log *ε*) 207 (3.45), 261 (3.68), 272 (3.65); ^1^H and ^13^C NMR data see Table 2; ^1^H-^1^H COSY and HMBC data see Supplementary Table S5; HRESIMS *m/z* 253.1799 (calcd for C_15_H_25_O_3_, 253.1798).

Compound **6**: colorless oil; [*α*]25 D +4 (*c* 0.10, MeOH); UV (MeOH) *λ*_max_ nm (log *ε*) 203 (3.80), 246 (3.64); ^1^H and ^13^C NMR data see Table 2; ^1^H-^1^H COSY and HMBC data see Supplementary Table S6; HRESIMS *m/z* 317.1728 (calcd for C_17_H_26_O_4_Na, 317.1723).

Compound **7**: colorless oil; [*α*]25 D -4 (*c* 0.05, MeOH); UV (MeOH) *λ*_max_ nm (log *ε*) 200 (3.91), 267 (3.53); ^1^H and ^13^C NMR data see Supplementary Table S7; HRESIMS *m/z* 237.1855 (calcd for C_15_H_25_O_2_, 237.2849).

Compound **8**: colorless oil; [*α*]25 D -20 (*c* 0.10, MeOH); UV (MeOH) *λ*_max_ nm (log *ε*) 204 (3.36), 243 (3.66), 248 (3.66); ^1^H and ^13^C NMR data see Supplementary Table S7; HRESIMS *m/z* 275.1616 (calcd for C_15_H_25_O_3_Na, 275.1618).

Compound **9**: colorless oil; [*α*]25 D +63.99 (*c* 0.10, MeOH); UV (MeOH) *λ*_max_ nm (log *ε*) 207 (3.53), 275 (2.96); ^1^H and ^13^C NMR data see Supplementary Table S7; HRESIMS *m/z* 309.2058 (calcd for C_18_H_29_O_4_, 309.2060).

Compound **10**: colorless oil; [*α*]25 D +15 (*c* 0.10, MeOH); UV (MeOH) *λ*_max_ nm (log *ε*) 205 (3.80); ^1^H/^13^C NMR and 2D NMR data see Supplementary Table S8; HRESIMS *m/z* 261.1831 (calcd for C_15_H_26_O_2_Na, 261.1825).

**Supplementary figures**


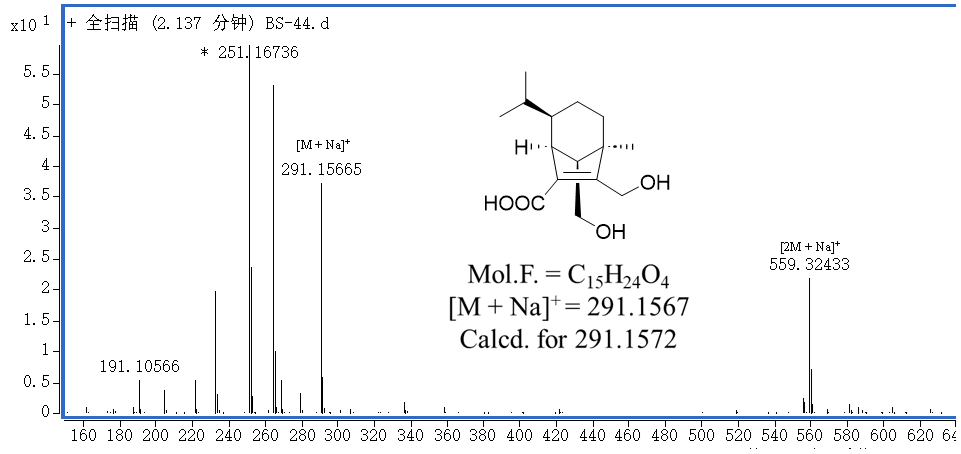


**Fig. S1a** HRESIMS spectrum of compound **1** in MeOH


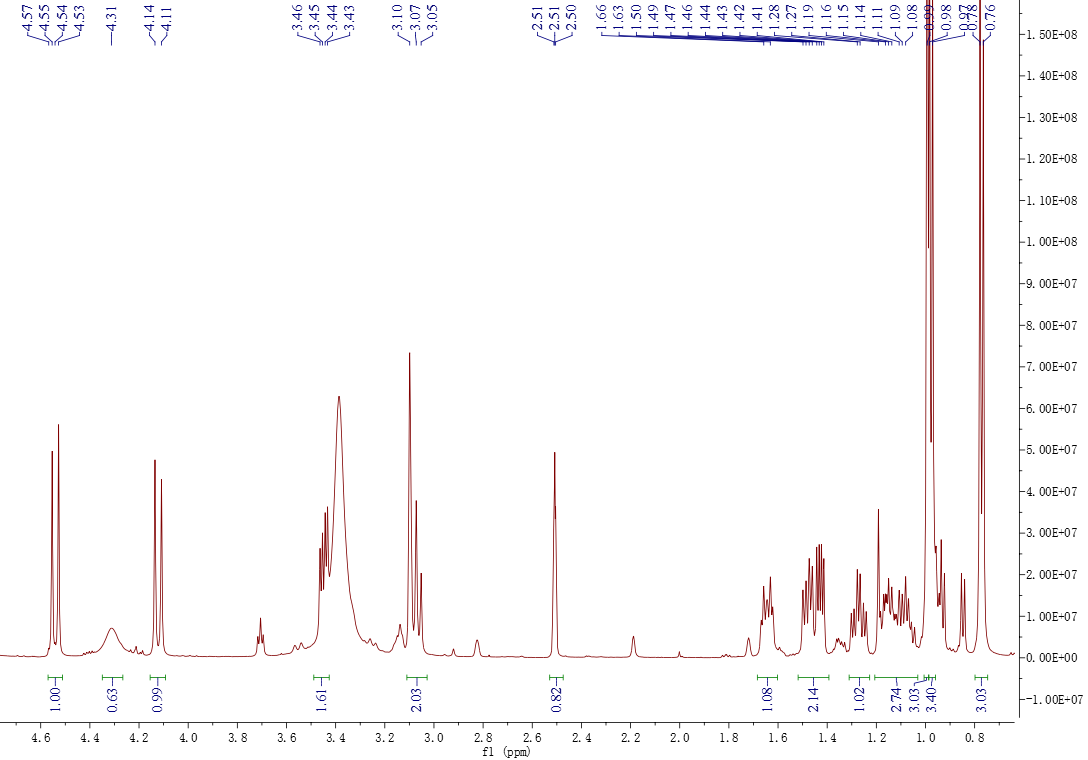


**Fig. S1b** ^1^H NMR (500 MHz, DMSO-*d_6_*) spectra of compound **1**


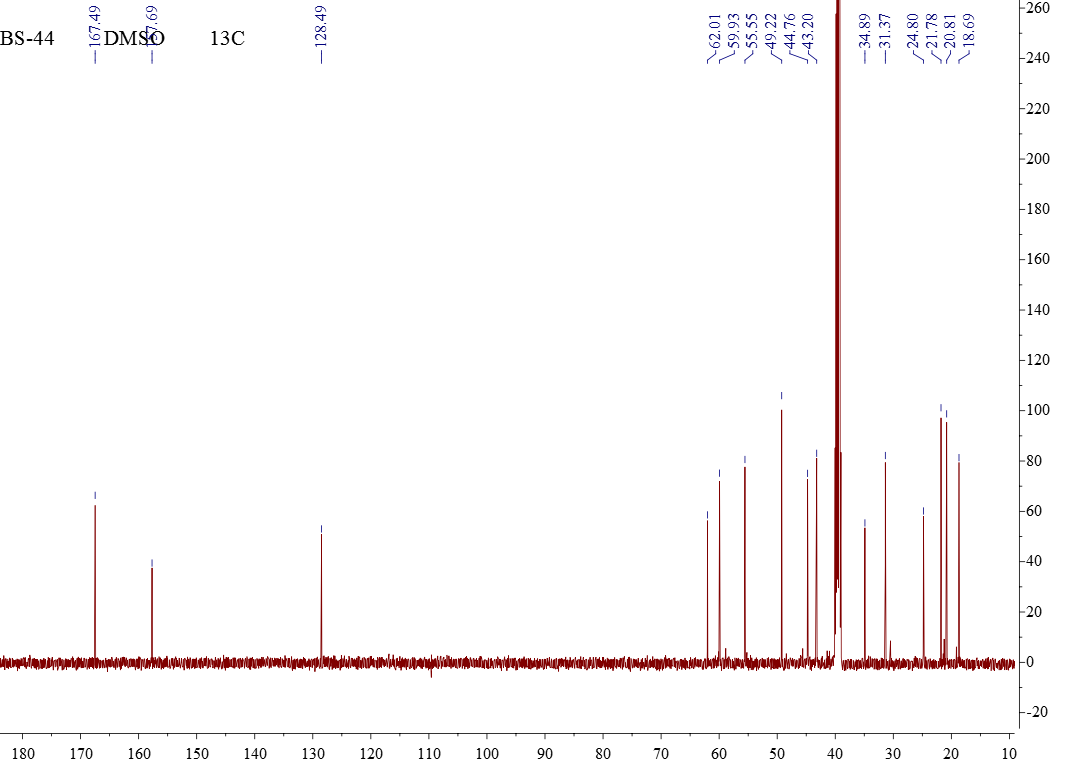


**Fig. S1c** ^13^C NMR (125 MHz, DMSO-*d_6_*) spectrum of compound **1**

**
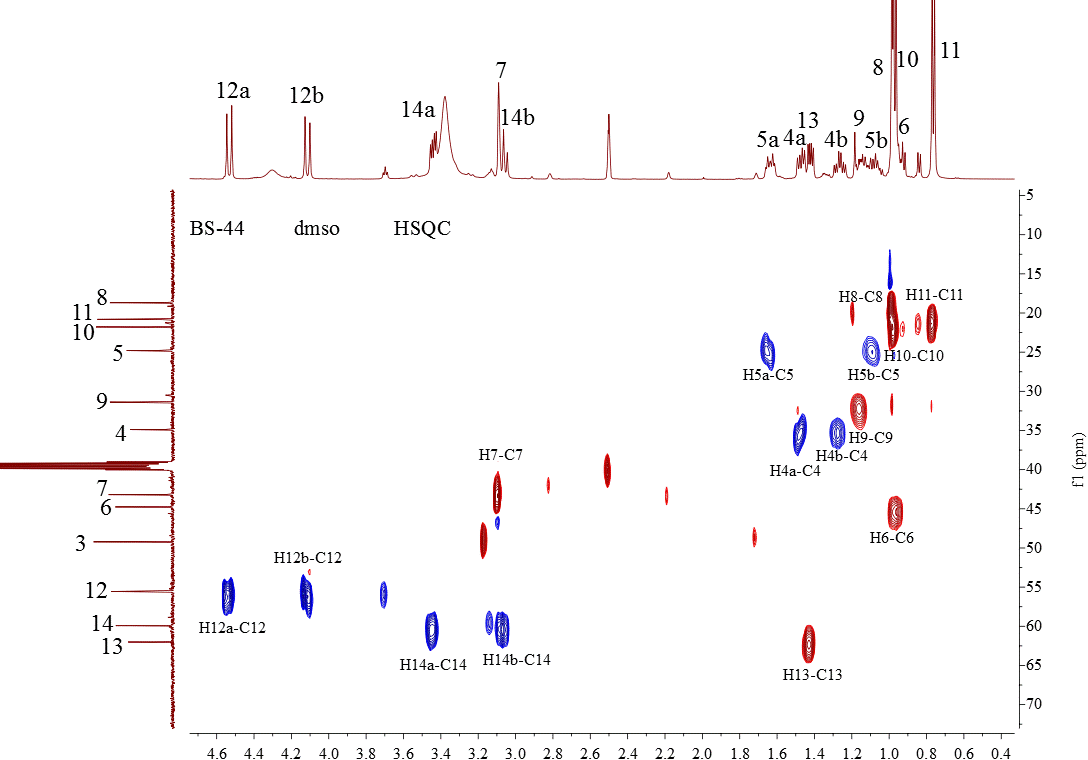
**

**Fig. S1d** HSQC (500 MHz, DMSO-*d_6_*) spectrum of compound **1**


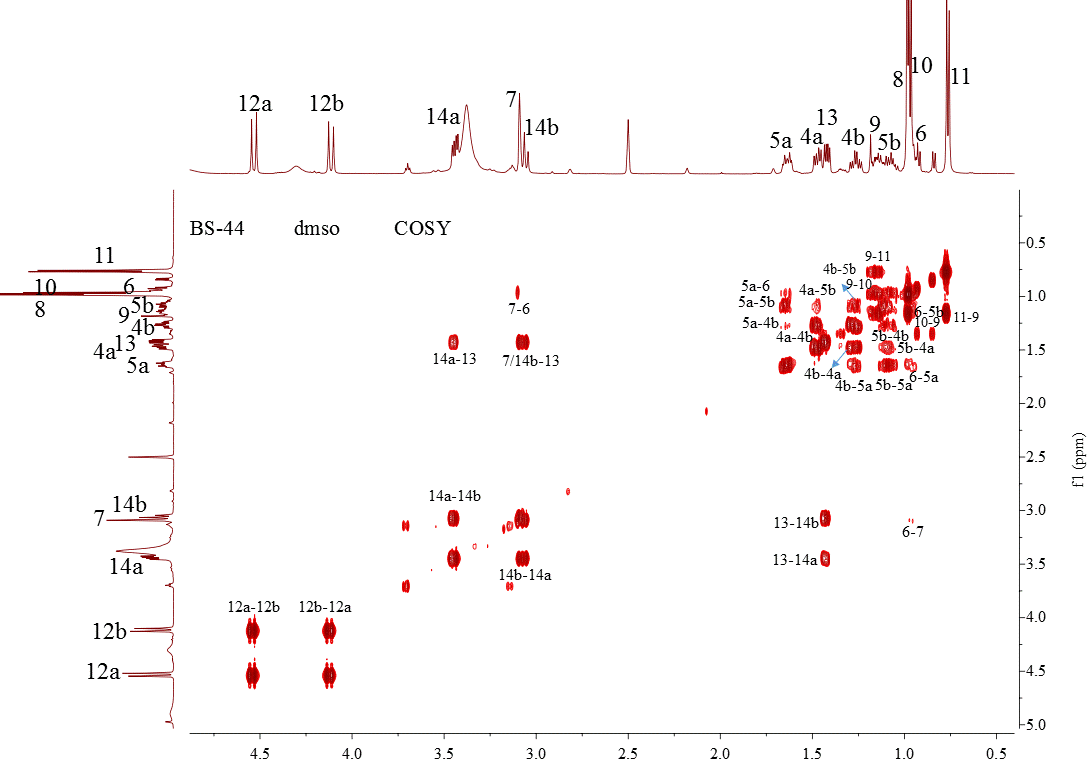


**Fig. S1e** ^1^H-^1^H COSY (500 MHz, DMSO-*d_6_*) spectrum of compound **1**

**
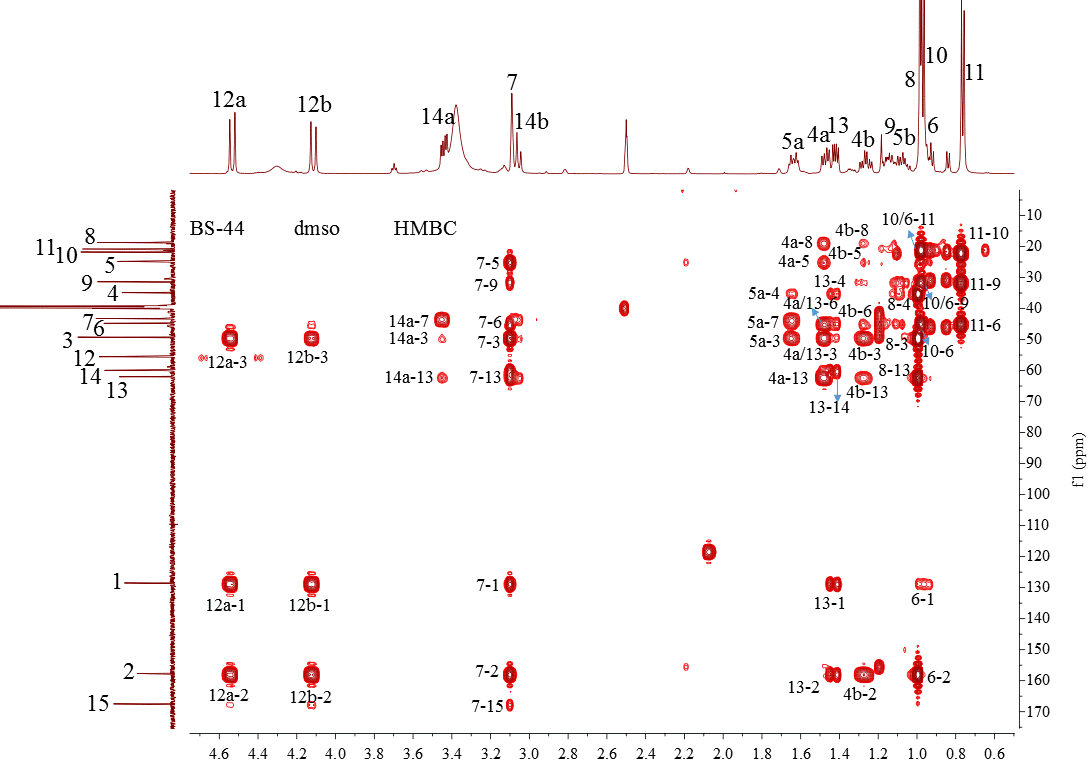
**

**Fig. S1f** HMBC (500 MHz, DMSO-*d_6_*) spectrum of compound **1**

**
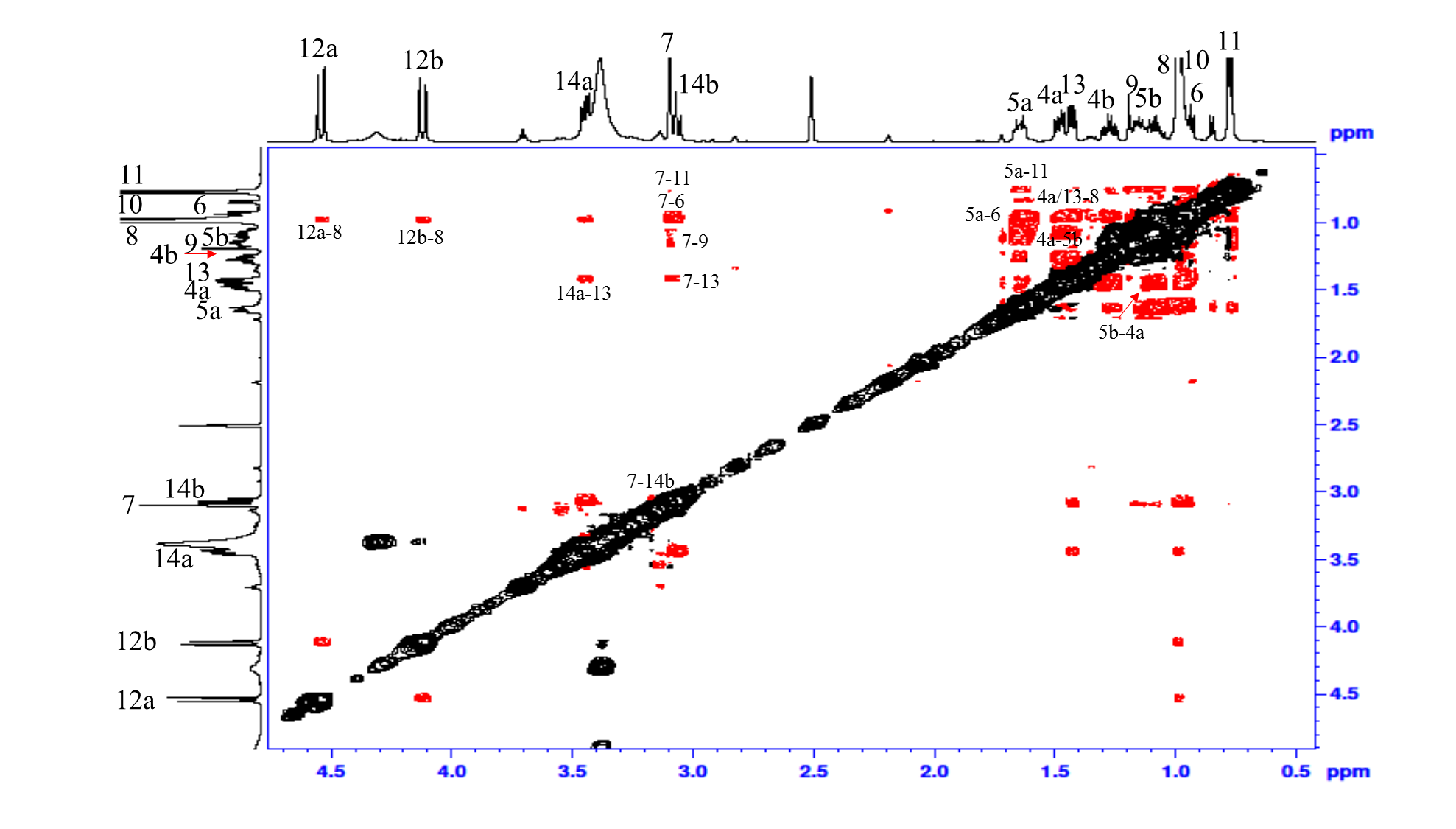
**

**Fig. S1g** ROESY (500 MHz, DMSO-*d_6_*) spectrum of compound **1**

**Table S1** NMR data of compound **1**

| Position | *δ*_C_, mult | *δ*_H_, mult (*J* in Hz) | COSY | HMBC | ROESY | Note |
| --- | --- | --- | --- | --- | --- | --- |
| 1 | 128.5, C |  |  |  |  |  |
| 2 | 157.7, C |  |  |  |  |  |
| 3 | 49.2, C |  |  |  |  |  |
| 4 | 34.9, CH2 | 1.48 dd (12.6, 6.2) | 4b, 5 | 3, 5, 6, 8, 13 | 8, 5 | a |
|  |  | 1.26 td (12.6, 5.7) | 4a, 5 | 2, 3, 5, 6, 8, 13 | 8, 5 | b |
| 5 | 24.8, CH2 | 1.64 m | 4, 5b, 6 | 3, 4, 7 | 6, 11 | a |
|  |  | 1.09 m | 4, 5a, 6 | 4, 6, 9 | 4 | b |
| 6 | 44.8, CH | 0.97 m | 5, 7, 9 | 1, 9, 11 | 5, 7, 11, 13 |  |
| 7 | 43.2, CH | 3.09 brs | 6, 13 | 1, 2, 5, 6, 9, 13, 15 | 6, 9, 11, 13, 14 |  |
| 8 | 18.7, CH3 | 0.99 s |  | 2, 3, 4, 13 | 4, 13, 14 |  |
| 9 | 31.4, CH | 1.16 m | 10, 11 | 5, 6, 11 | 7, 10, 11 |  |
| 10 | 21.8, CH3 | 0.97 d (6.3) | 9 | 6, 9, 11 | 9, 11 |  |
| 11 | 20.8, CH3 | 0.76 d (6.3) | 9 | 6, 9, 10 | 5, 6, 7, 9, 10 |  |
| 12 | 55.6, CH2 | 4.54 d (13.2) | 12b | 1, 2, 3 | 8 | a |
|  |  | 4.12 d (13.2) | 12a | 1, 2, 3 | 8 | b |
| 13 | 62.0, CH | 1.42 dd (9.4, 4.9) | 7, 14 | 1, 2, 3, 4, 6, 14 | 6, 7, 8, 14 |  |
| 14 | 59.9, CH2 | 3.44 dd (10.3, 4.9) | 13 | 3, 7, 13 | 13 | a |
|  |  | 3.05 d (10.3) | 13 | 7, 13 | 13 | b |
| 15 | 167.5, C |  |  |  |  |  |

**
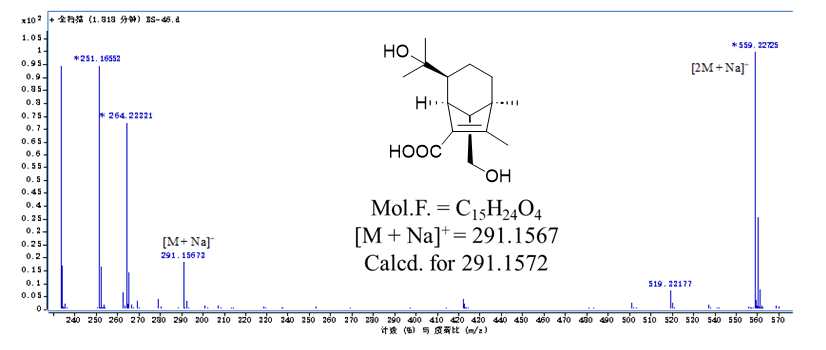
**

**Fig. S2a** HRESIMS spectrum of compound **2** in MeOH


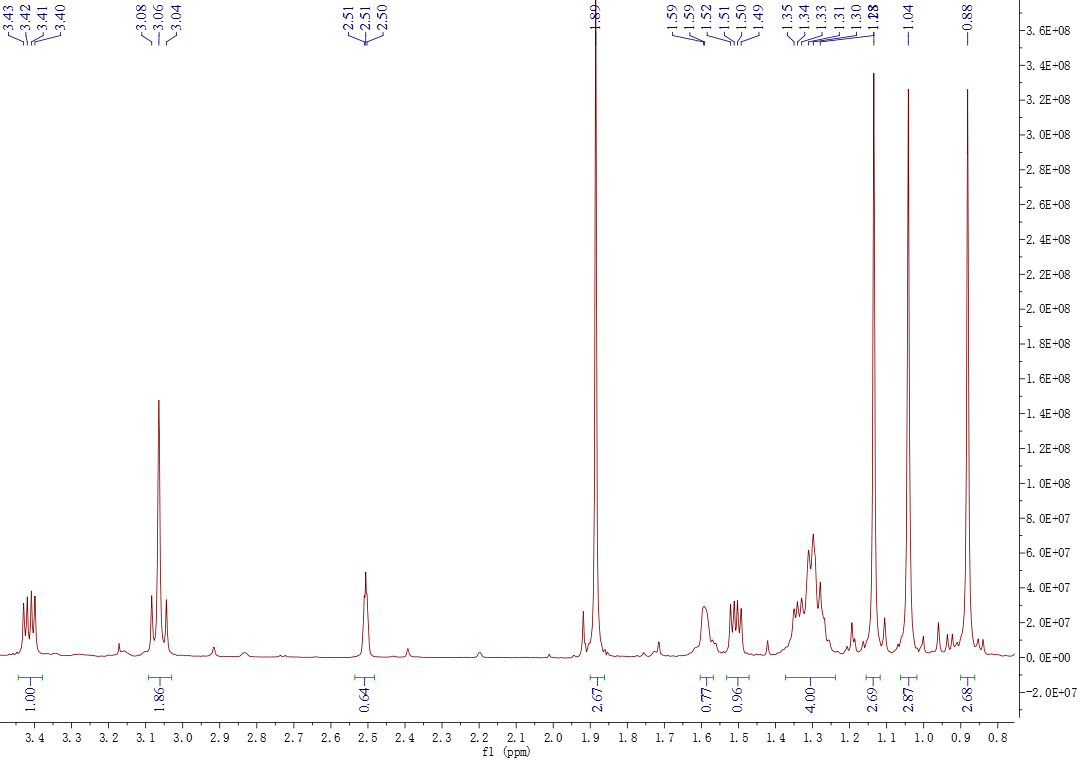


**Fig. S2b** ^1^H NMR (500 MHz, DMSO-*d_6_*) spectra of compound **2**


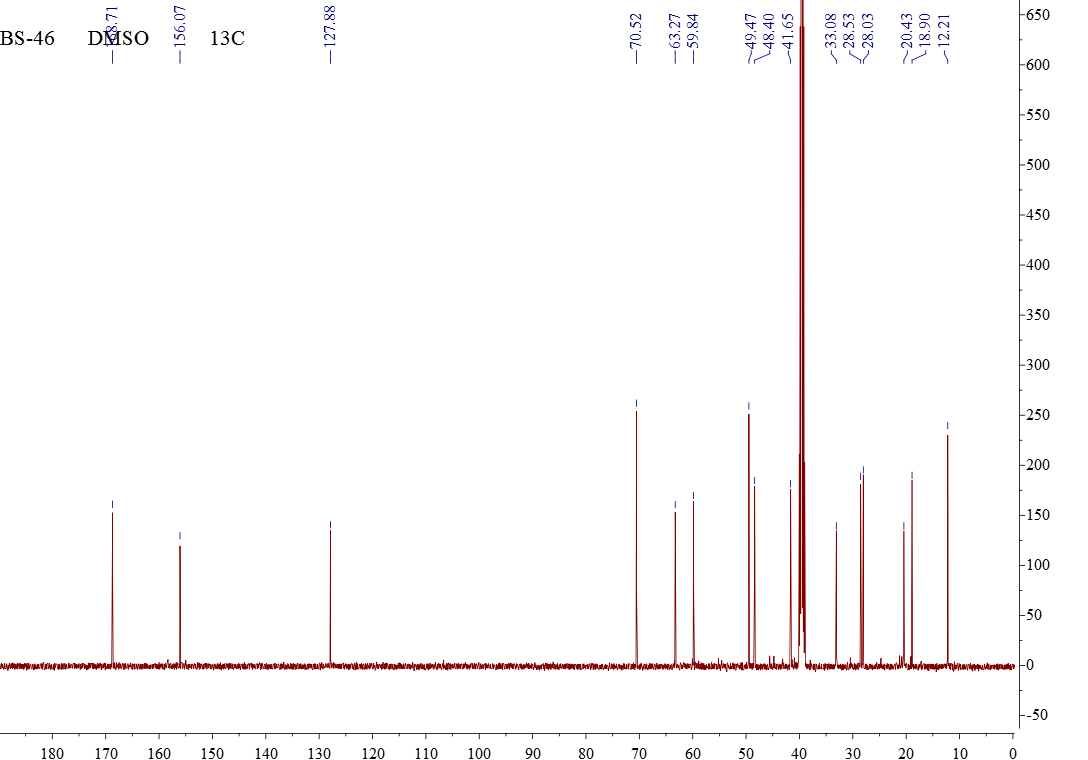


**Fig. S2c** ^13^C NMR (125 MHz, DMSO-*d_6_*) spectrum of compound **2**

**
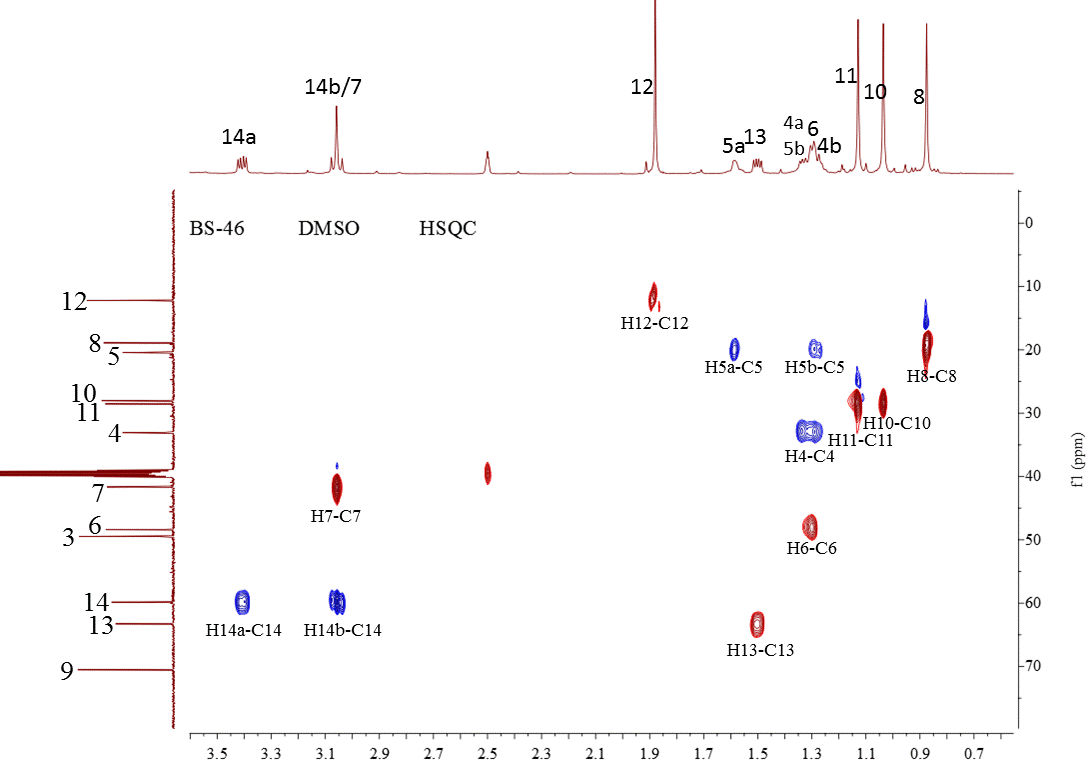
**

**Fig. S2d** HSQC (500 MHz, DMSO-*d_6_*) spectrum of compound **2**


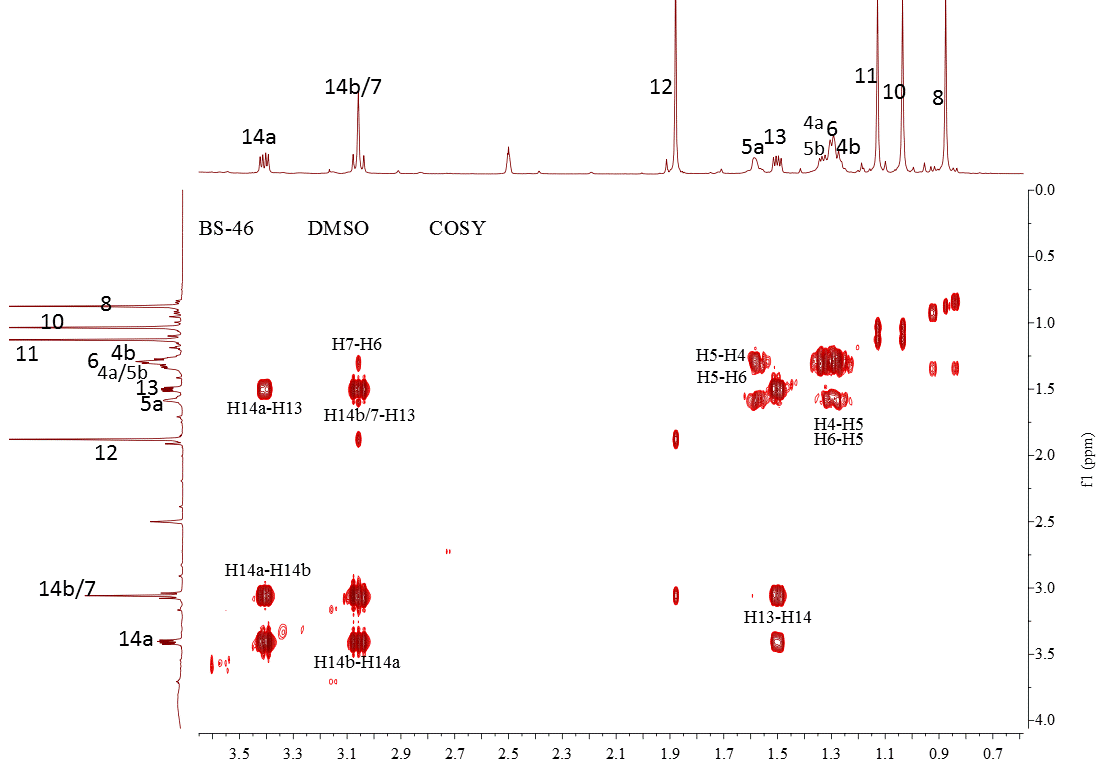


**Fig. S2e** ^1^H-^1^H COSY (500 MHz, DMSO-*d_6_*) spectrum of compound **2**


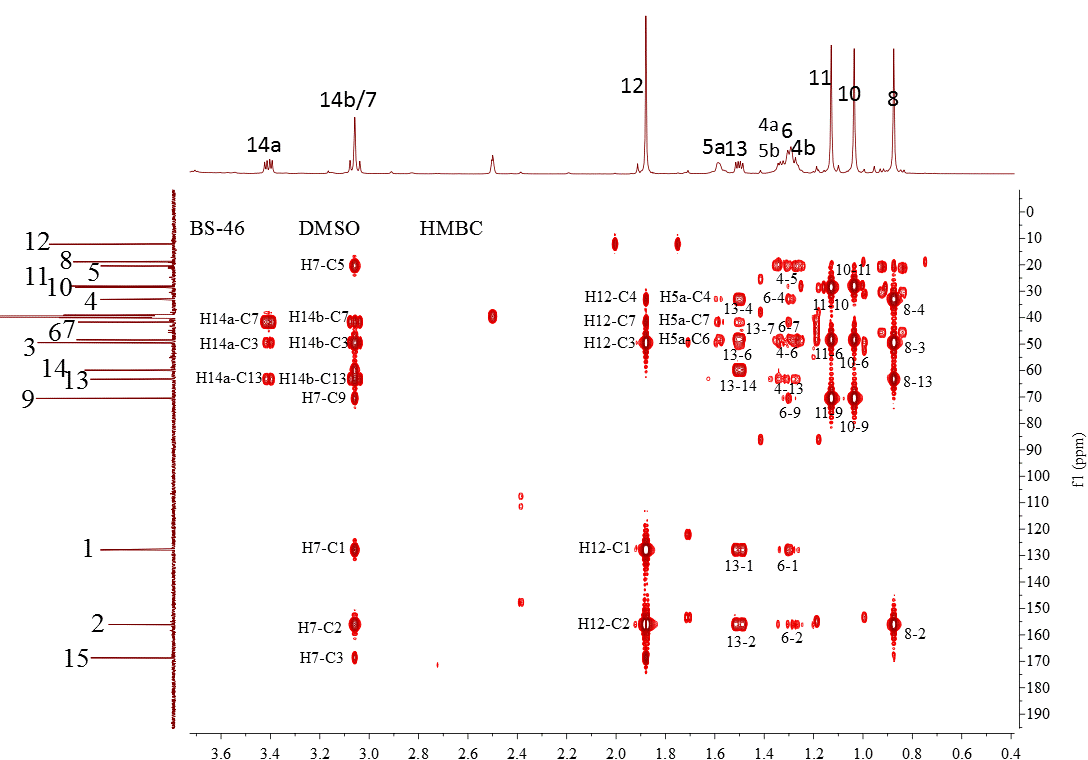


**Fig. S2f** HMBC (500 MHz, DMSO-*d_6_*) spectrum of compound **2**


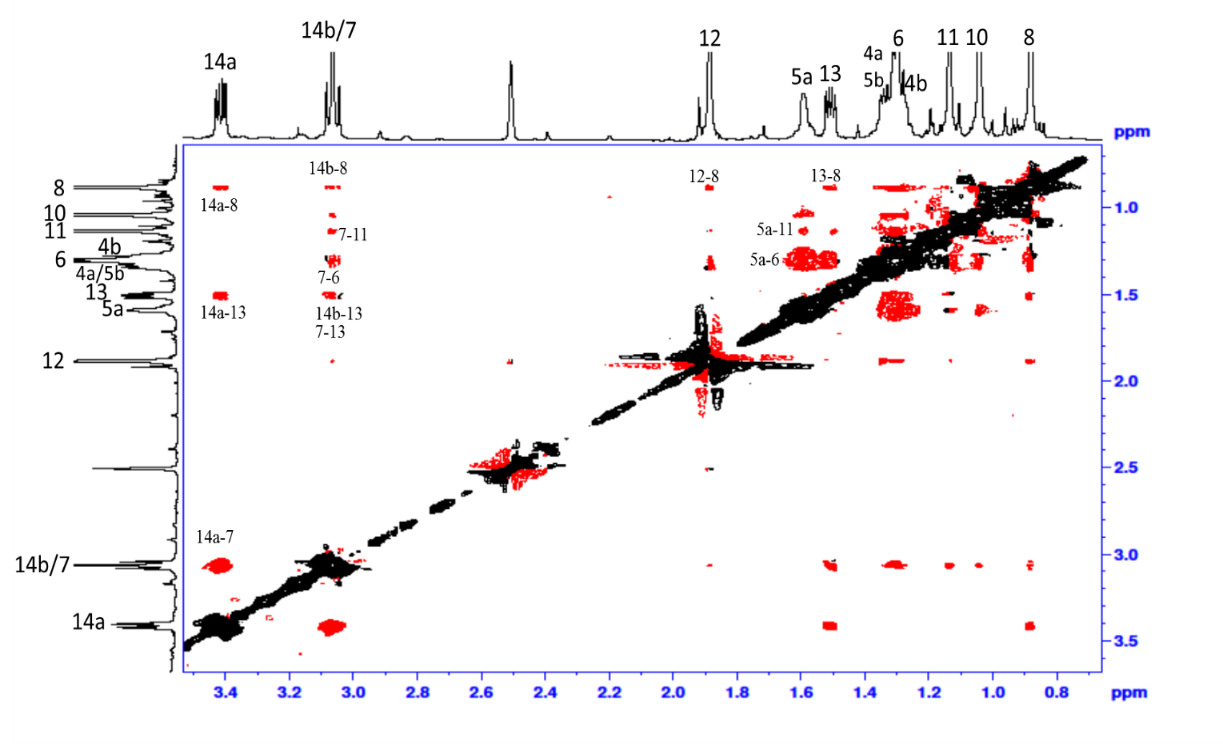


**Fig. S2g** ROESY (500 MHz, DMSO-*d_6_*) spectrum of compound **2**

**Table S2** NMR data of compound **2**

| Position | *δ*_C_, mult | *δ*_H_, mult (*J* in Hz) | COSY | HMBC | ROESY | note |
| --- | --- | --- | --- | --- | --- | --- |
| 1 | 127.9, C |  |  |  |  |  |
| 2 | 156.1, C |  |  |  |  |  |
| 3 | 49.5, C |  |  |  |  |  |
| 4 | 33.1, CH_2_ | 1.33 m | 5 | 2, 5, 8, 13 | 5 | a |
|  |  | 1.28 m | 5 | 2, 5, 8, 13 | 5 | b |
| 5 | 20.4, CH_2_ | 1.59 m | 4, 6 | 3, 4, 6, 7 | 6, 11 | a |
|  |  | 1.28 m | 4, 6 | 4, 6, 9 | 6 | b |
| 6 | 48.4, CH | 1.30 m | 5, 7 | 1, 4, 5, 7, 9, 13 | 5, 7 |  |
| 7 | 41.7, CH | 3.06 brs | 6, 13 | 1, 2, 5, 14, 15 | 6, 11, 13, 14 |  |
| 8 | 18.9, CH_3_ | 0.88 s |  | 2, 3, 4, 13 | 12, 13, 14 |  |
| 9 | 70.5, C |  |  |  |  |  |
| 10 | 28.5, CH_3_ | 1.04 s |  | 6, 9, 11 | 6 |  |
| 11 | 28.0, CH_3_ | 1.13 s |  | 6, 9, 10 | 5, 7 |  |
| 12 | 12.2, CH_3_ | 1.88 s |  | 1, 2, 3 | 8 |  |
| 13 | 63.3, CH | 1.50 dd (9.5, 5.2) | 7, 14 | 1, 2, 3, 4, 7, 14 |  |  |
| 14 | 59.8, CH_2_ | 3.40 dd (10.2, 5.2) | 13 | 3, 7, 13 | 8, 13 | a |
|  |  | 3.06 t (10.2) | 13 | 3, 7, 13 | 8, 13 | b |
| 15 | 168.7, C |  |  |  |  |  |

**Fig. S3a** HRESIMS spectrum of compound **3** in MeOH

**Fig. S3b** ^1^H NMR (800 MHz, DMSO-*d*_6_) spectra of compound **3**

**Fig. S3c** ^13^C NMR (200 MHz, DMSO-*d*_6_) spectrum of compound **3**
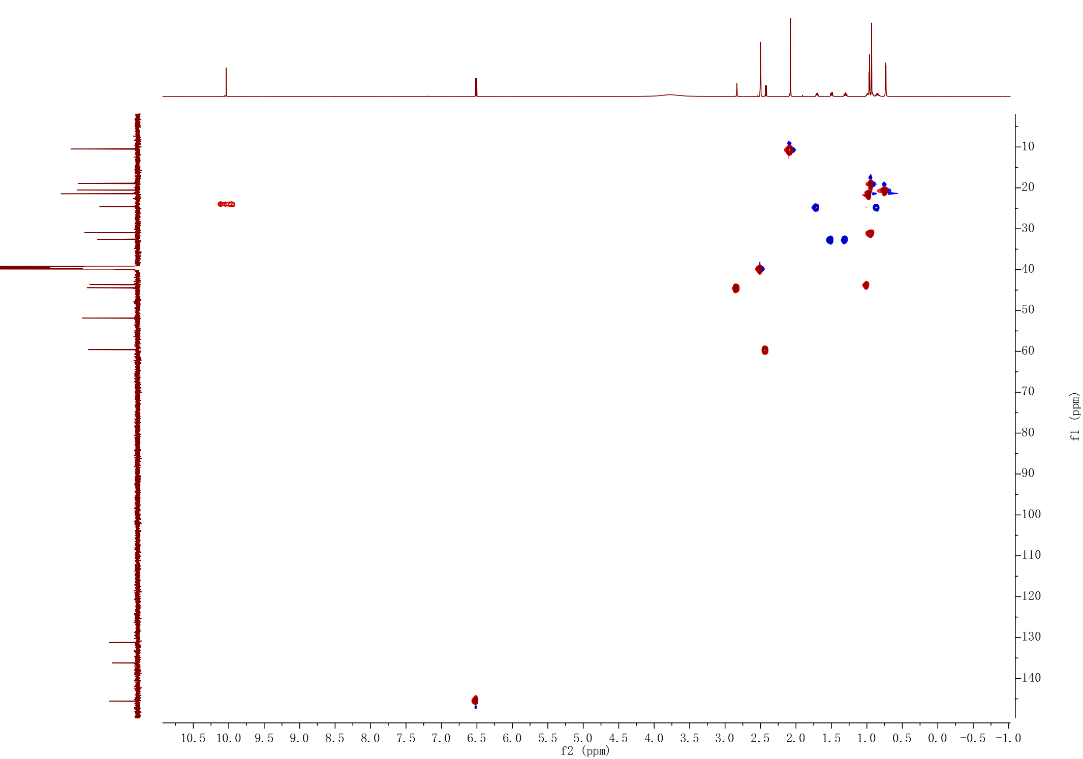


**Fig.** **S3d** HSQC (800 MHz, DMSO-*d*_6_) spectrum of compound **3**

**Fig. S3e** ^1^H-^1^H COSY (800 MHz, DMSO-*d*_6_) spectrum of compound **3**

**Fig.** **S3f** HMBC (800 MHz, DMSO-*d*_6_) spectrum of compound **3**

**Fig. S3g** ROESY (800 MHz, DMSO-*d*_6_) spectrum of compound **3**

**Table S3** NMR data of compound **3**

| Position | *δ*_C_, mult | *δ*_H_, mult (*J* in Hz) | HMBC | ROESY |
| --- | --- | --- | --- | --- |
| 1 | 136.2, C |  |  |  |
| 2 | 165.7, C |  |  |  |
| 3 | 51.8, C |  |  |  |
| 4 | 32.6, CH_2_ | 1.50 m | 8, 5, 6, 3, 13 | 5b, 10, 4b, |
|  |  | 1.30 m |  | 5b, 10 |
| 5 | 24.6, CH_2_ | 1.70 m | 4, 7, 3 | 11, 5b, 10, 4 |
|  |  | 0.85 m |  |  |
| 6 | 43.7, CH | 1.00 m | 8, 9 |  |
| 7 | 44.4, CH | 2.83 s | 5, 9, 6, 3, 13, 1, 14, 2, 15 | 11, 10, 13 |
| 8 | 18.9, CH_3_ | 0.93 s | 4, 3, 13, 2 |  |
| 9 | 31.0, CH | 0.94 m | 5, 7 |  |
| 10 | 21.5, CH_3_ | 0.97 d (6.0) | 11, 9, 6 |  |
| 11 | 20.5, CH_3_ | 0.73 d (6.0) | 10, 9, 6 |  |
| 12 | 10.5, CH_3_ | 2.08 s | 3, 1, 2 | 8, 4a |
| 13 | 59.6, CH | 2.42 d (10.9) | 4, 6, 3, 16, 1, 14, 2 | 10, 4b |
| 14 | 145.6, CH | 6.51 d (10.9) | 7, 3, 16, 17, 18 | 9, 13, 7 |
| 15 | 188.6, CH | 10.03 s | 7, 1 |  |
| 16 | 131.2, C |  |  |  |
| 17 | 164.9, C |  |  |  |
| 18 | 166.8, C |  |  |  |

**Fig. S4a** HRESIMS spectrum of compound **4** in MeOH

**Fig. S4b** ^1^H NMR (800 MHz, DMSO-*d*_6_) spectra of compound **4**

**Fig. S4c** ^13^C NMR (200 MHz, DMSO-*d*_6_) spectrum of compound **4**

**Fig.** **S4d** HSQC (800 MHz, DMSO-*d*_6_) spectrum of compound **4**

**Fig. S4e** ^1^H-^1^H COSY (800 MHz, DMSO-*d*_6_) spectrum of compound **4****Fig.** **S4f** HMBC (800 MHz, DMSO-*d*_6_) spectrum of compound **4**

**Fig. S4g** ROESY (800 MHz, DMSO-*d*_6_) spectrum of compound **4**

**Table S4** NMR data of compound **4**

| Position | *δ*_C_, mult | *δ*_H_, mult (*J* in Hz) | HMBC | ROESY |
| --- | --- | --- | --- | --- |
| 1 | 136.0, C |  |  |  |
| 2 | 166.0, C |  |  |  |
| 3 | 50.4, C |  |  |  |
| 4 | 33.1, CH_2_ | 1.39 m | 6, 13 | 5b, 8, 11 |
|  |  |  |  |  |
| 5 | 24.7, CH_2_ | 1.66 m |  | 5b, 8, 11, 4 |
|  |  | 0.81 m |  |  |
| 6 | 44.1, CH | 0.98 m |  |  |
| 7 | 41.0, CH | 2.94 s | 5, 3, 13, 14, 1, 2 | 10, 11, 8, 13,14b |
| 8 | 18.0, CH_3_ | 1.00 s | 4, 3, 13 |  |
| 9 | 31.1, CH | 0.94 m |  |  |
| 10 | 20.5, CH_3_ | 0.73 d (6.5) | 11, 9, 6 |  |
| 11 | 21.5, CH_3_ | 0.99 d (7.0) | 10, 9, 6 |  |
| 12 | 10.3, CH_3_ | 2.03 s | 3, 1, 2 | 8, 11 |
| 13 | 56.9, CH | 1.75 dd (9.0, 5.5) | 14, 1, 2 | 8, 11, 4 |
| 14 | 63.6, CH_2_ | 4.00 dd (11.1, 5.5) |  | 8, 11, 13 |
|  |  | 3.66 dd (11.1, 8.9) | 7, 3, 13, 16 | 13, 7 |
| 15 | 188.4, CH | 9.99 s | 7, 1 |  |
| 16 | 170.4, C |  |  |  |
| 17 | 20.7, CH_3_ | 1.98 s | 16 |  |

**
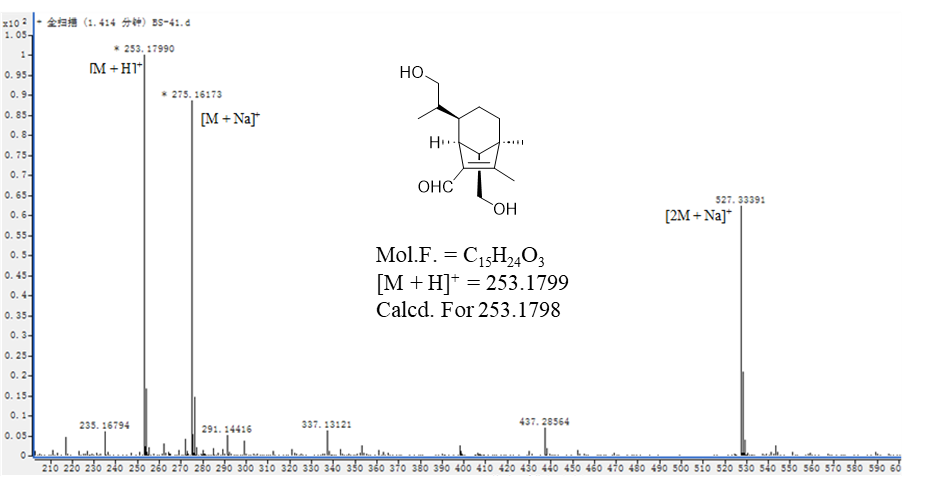
**

**Fig. S5a** HRESIMS spectrum of compound **5** in MeOH


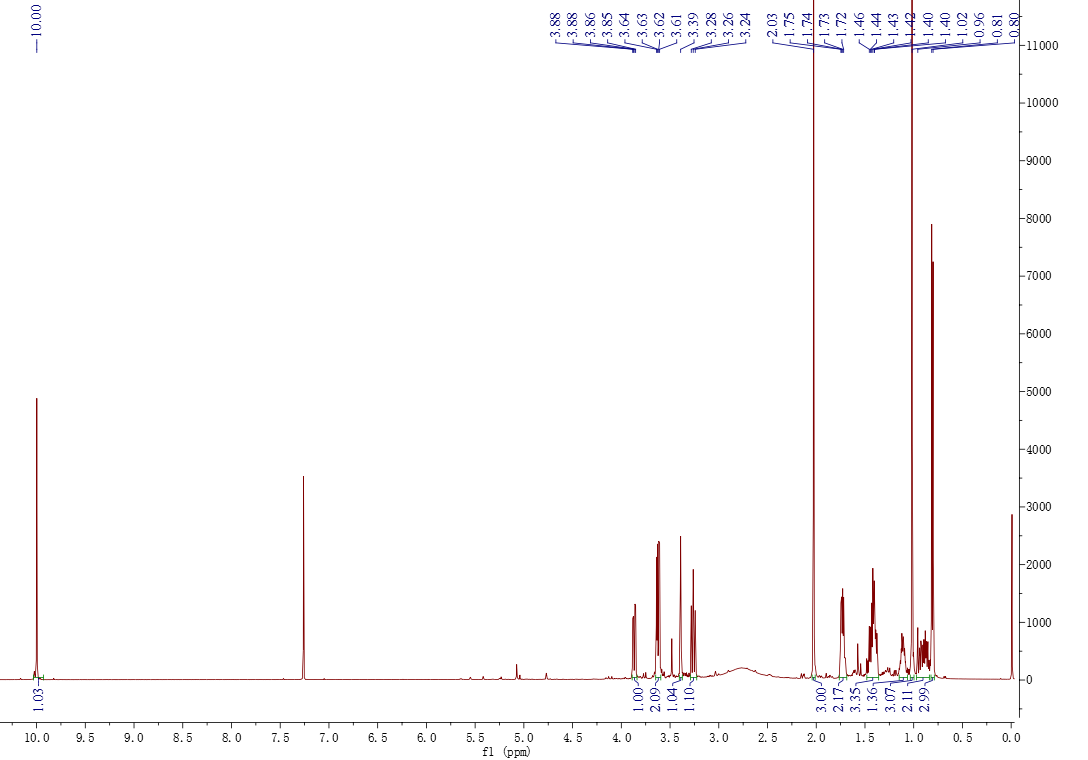


**Fig. S5b** ^1^H NMR (500 MHz, CDCl3) spectra of compound **5**


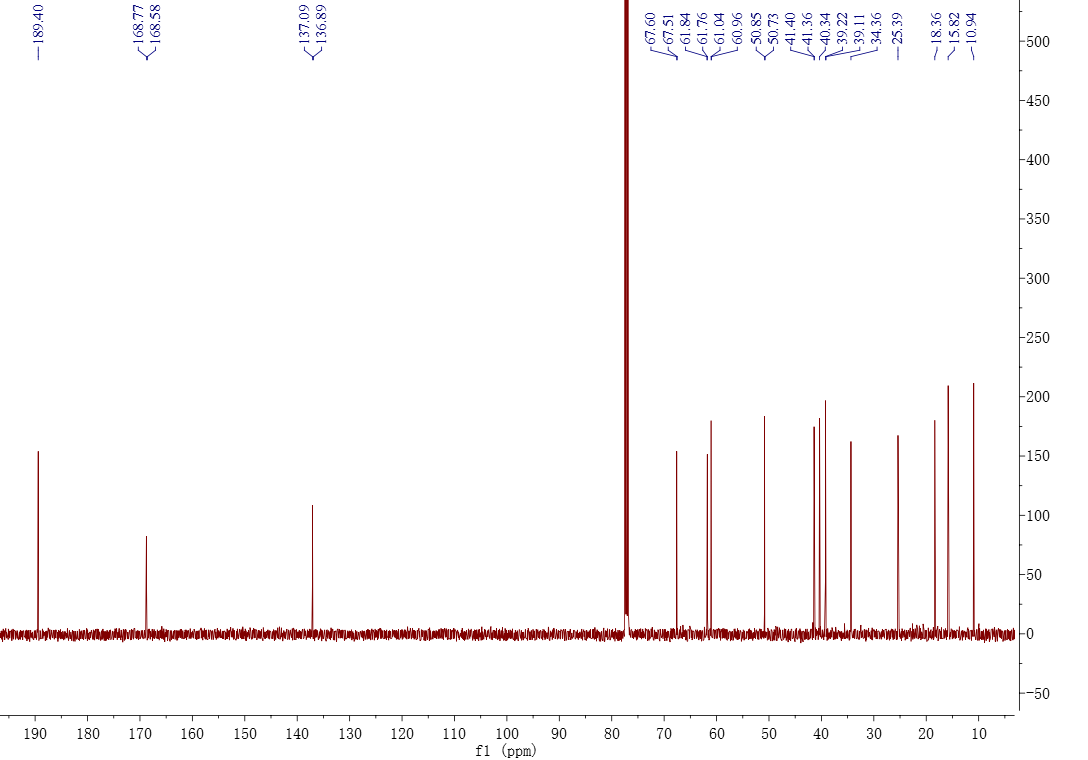


**Fig. S5c** ^13^C NMR (125 MHz, CDCl3) spectrum of compound **5**


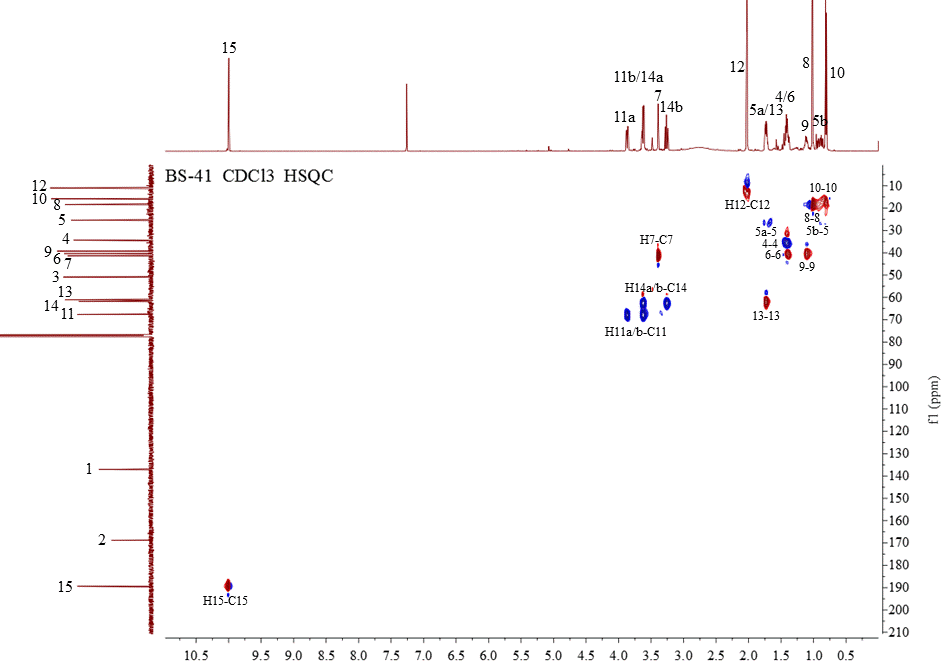


**Fig. S5d** HSQC (500 MHz, CDCl3) spectrum of compound **5**

**
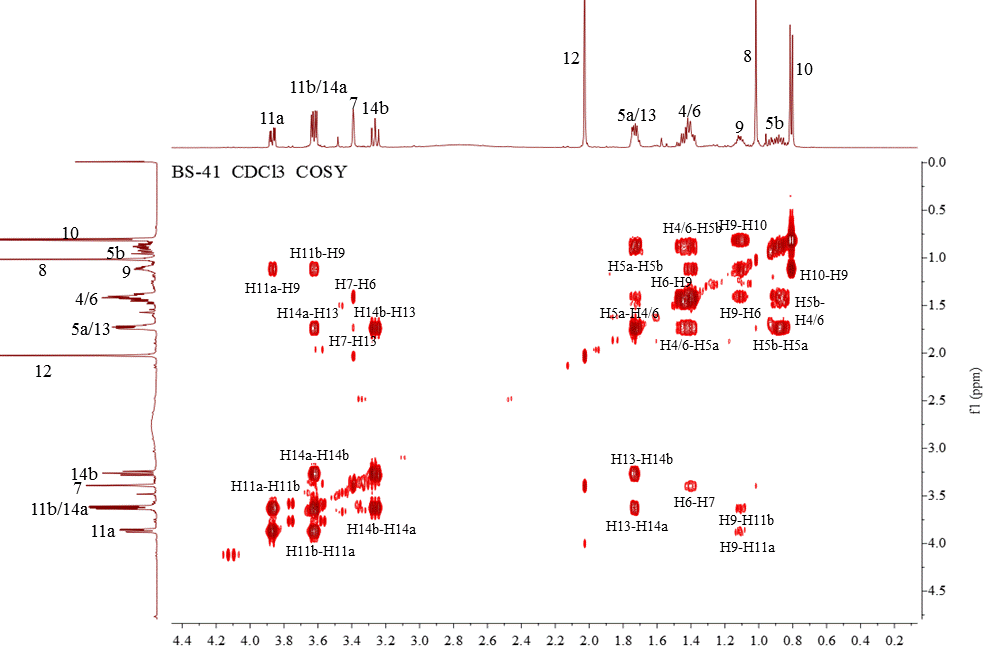
**

**Fig. S5e** ^1^H-^1^H COSY (500 MHz, CDCl3) spectrum of compound **5**


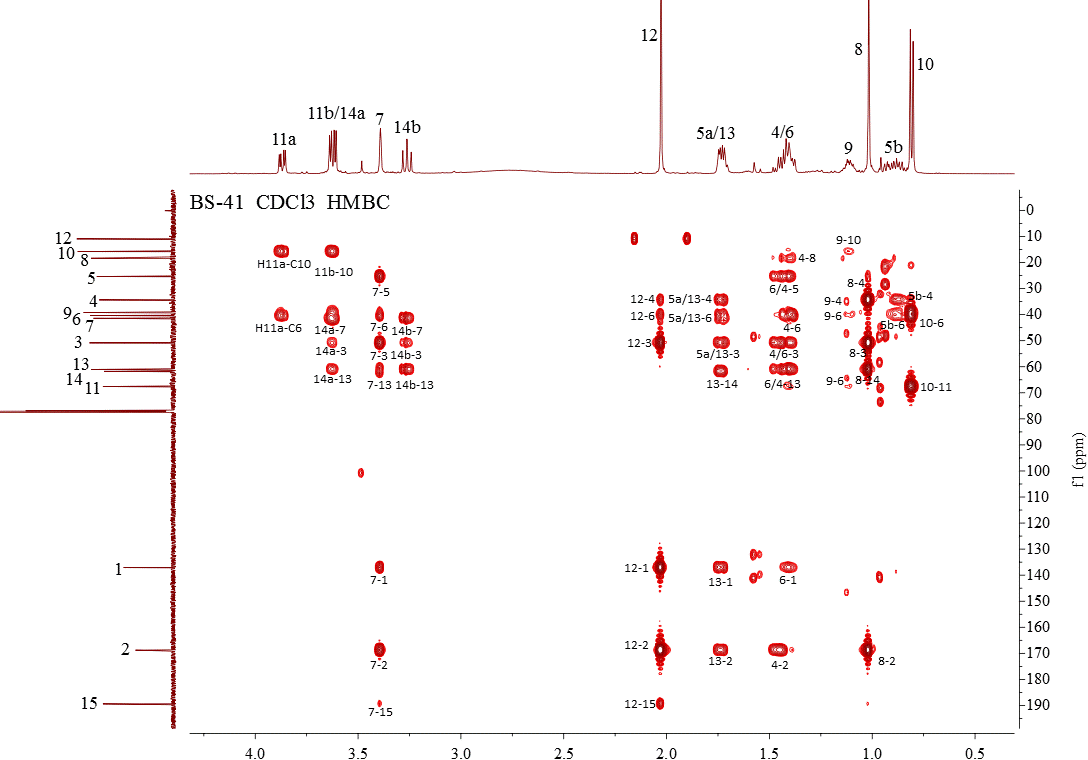


**Fig. S5f** HMBC (500 MHz, CDCl3) spectrum of compound **5**


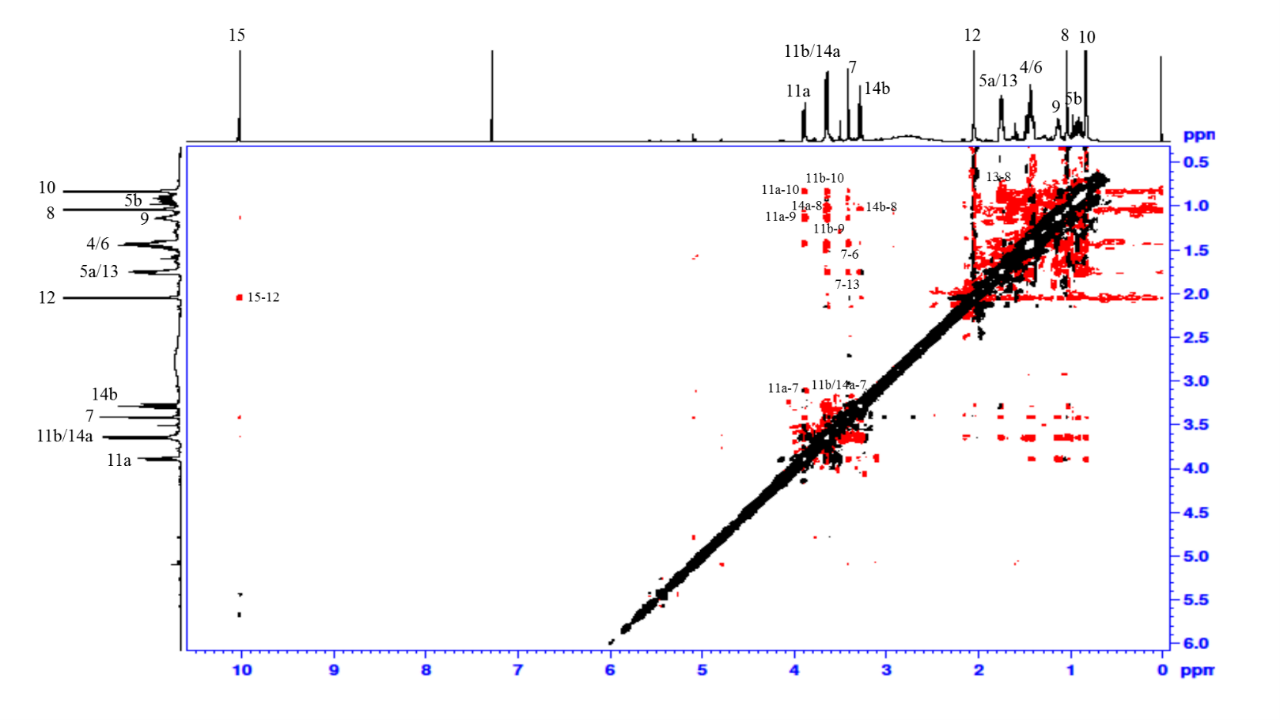


**Fig. S5g** ROESY (500 MHz, CDCl3) spectrum of compound **5**

**Table S5** NMR data of compound **5**

| Position | *δ*_C_, mult | *δ*_H_, mult (*J* in Hz) | COSY | HMBC | ROESY |
| --- | --- | --- | --- | --- | --- |
| 1 | 137.1, C |  |  |  |  |
| 2 | 168.7, C |  |  |  |  |
| 3 | 50.8, C |  |  |  |  |
| 4 | 34.3, CH_2_ | 1.43 m | 5 | 2, 3, 5, 6, 8, 13 | 8 |
| 5 | 25.4, CH_2_ | 1.73 m | 4, 6 | 3, 4, 6 | 6 |
|  |  | 0.9 m | 4, 6 | 4, 6 |  |
| 6 | 40.3, CH | 1.43 m | 5, 7, 9 | 1, 5, 13 | 5, 7, 13 |
| 7 | 41.4, CH | 3.39 brs | 6, 13 | 1, 2, 5, 6, 13, 15 | 6, 11, 13, 14 |
| 8 | 18.4, CH_3_ | 1.02 s |  | 3, 4, 13 | 4, 13, 14 |
| 9 | 39.2, CH | 1.11 m | 6, 10, 11 | 6, 10, 11 | 11 |
| 10 | 15.8, CH_3_ | 0.80 d (6.9) | 9 | 6, 11 | 11 |
| 11 | 67.7, CH_2_ | 3.87 dd (11.1, 3.9) | 9 | 6, 10 | 7, 9, 10 |
|  |  | 3.62 dd (11.1, 4.7) | 9 | 10 | 7, 9, 10 |
| 12 | 10.9, CH_3_ | 2.03 s |  | 1, 2, 3 | 15 |
| 13 | 61.0, CH | 1.73 m | 7, 14 | 1, 3, 4, 6, 14 | 6, 7, 8 |
| 14 | 61.8, CH_2_ | 3.62 dd (11.1, 4.7) | 13 | 3, 7, 13 | 7, 8 |
|  |  | 3.26 t (11.1) | 13 | 3, 7, 13 | 7, 8 |
| 15 | 189.4, CH | 10.00 s |  | 1, 7 | 12 |

**
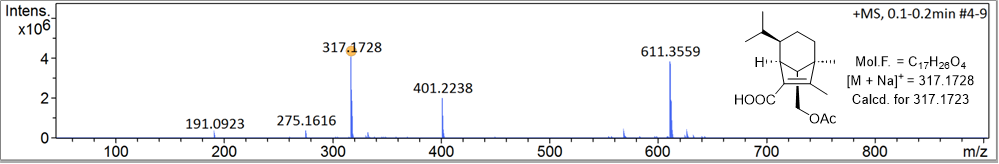
**

**Fig. S6a** HRESIMS spectrum of compound **6** in MeOH

**Fig. S6b** ^1^H NMR (800 MHz, DMSO-*d*_6_) spectra of compound **6**

**Fig. S6c** ^13^C NMR (200 MHz, DMSO-*d*_6_) spectrum of compound **6**

**Fig. S6d** HSQC (800 MHz, DMSO-*d*_6_) spectrum of compound **6**

**Fig. S6e** ^1^H-^1^H COSY (800 MHz, DMSO-*d*_6_) spectrum of compound **6**

**Fig. S6f** HMBC (800 MHz, DMSO-*d*_6_) spectrum of compound **6**

**Fig. S6g** ROESY (800 MHz, DMSO-*d*_6_) spectrum of compound **6**

**Table S6** NMR data of compound **6**

| Position | *δ*_C_, mult | *δ*_H_, mult (*J* in Hz) |
| --- | --- | --- |
| 1 | 126.5, C |  |
| 2 | 156.7, C |  |
| 3 | 49.6, C |  |
| 4 | 32.9, CH_2_ | 1.33 m |
|  |  | 1.29 m |
| 5 | 24.7, CH_2_ | 1.65 m |
|  |  | 0.87 m |
| 6 | 44.4, CH | 0.96 m |
| 7 | 43.3, CH | 2.91 s |
| 8 | 18.7, CH_3_ | 0.94 s |
| 9 | 31.1, CH | 1.12 m |
| 10 | 20.6, CH_3_ | 0.75 d (6.4) |
| 11 | 21.6, CH_3_ | 0.96 d (6.4) |
| 12 | 11.9, CH_3_ | 1.93 s |
| 13 | 57.4, CH | 1.68 dd (5.6, 8.8) |
| 14 | 63.9, CH_2_ | 4.02 dd (5.6, 10.9) |
|  |  | 3.71 dd (8.8, 10.9) |
| 15 | 167.9, C |  |
| 16 | 170.4, C |  |
| 17 | 20.8, CH_3_ | 1.99 s |


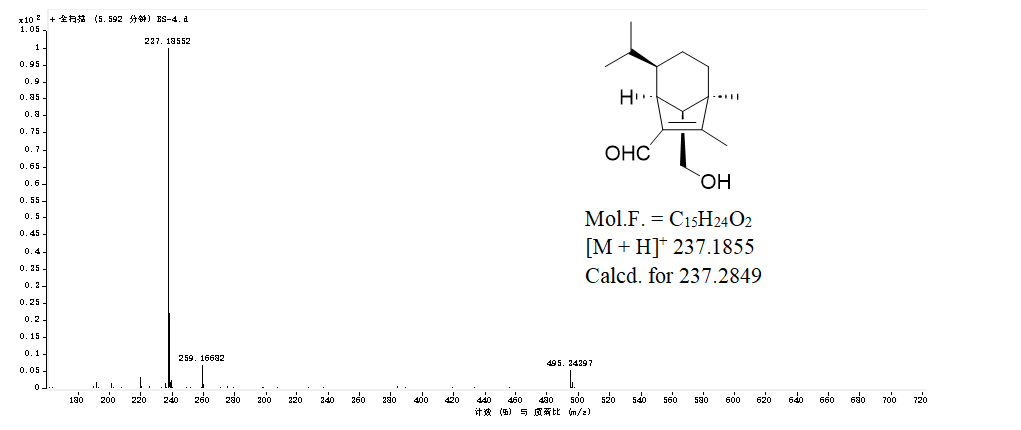


**Fig. S7a** HRESIMS spectrum of compound **7** in MeOH


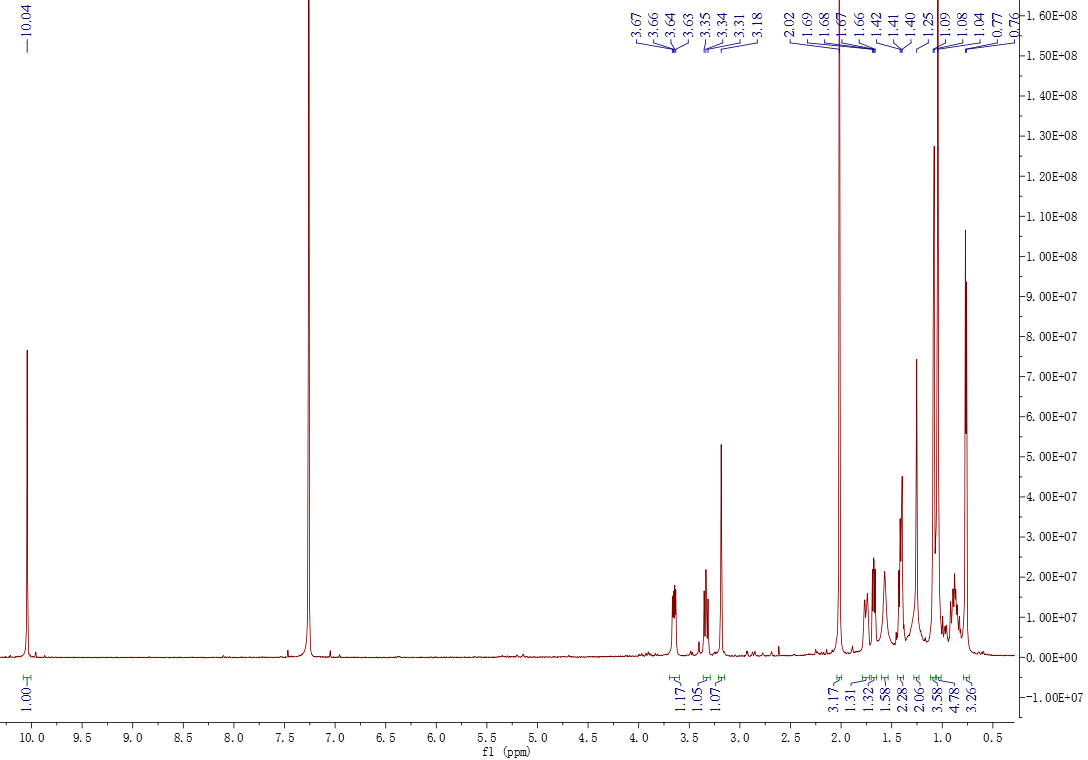


**Fig. S7b** ^1^H NMR (500 MHz, CDCl3) spectra of **7**


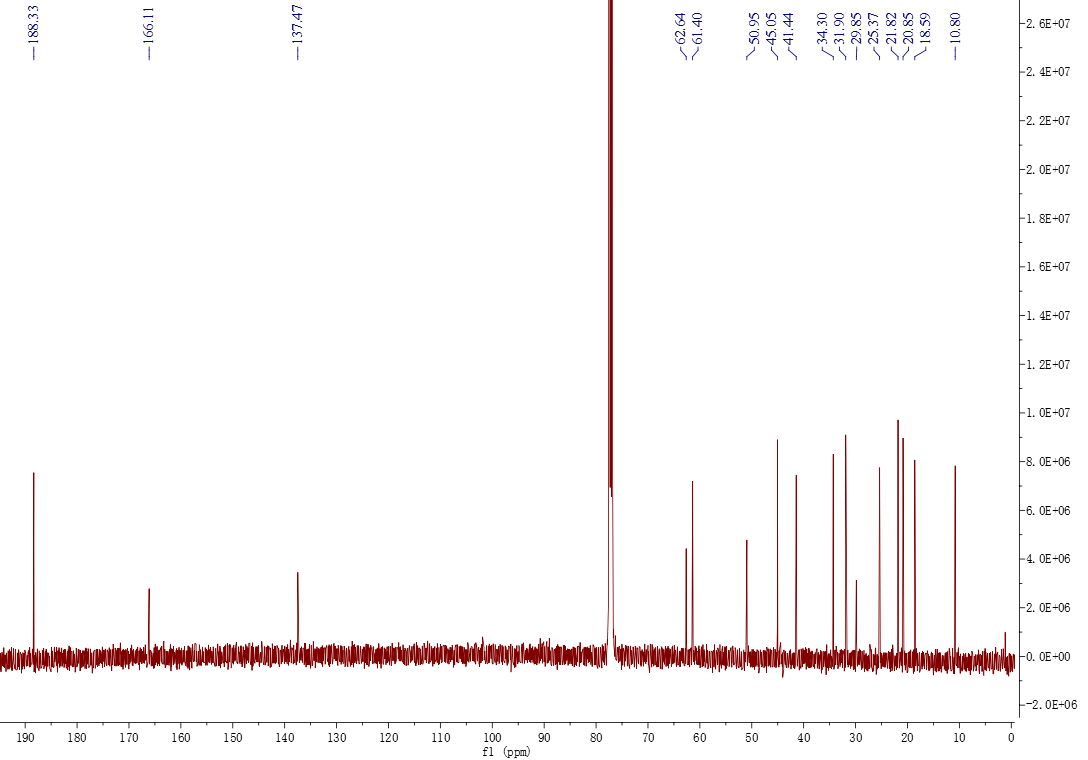


**Fig. S7c** ^13^C NMR (125 MHz, CDCl3) spectrum of **7**

**
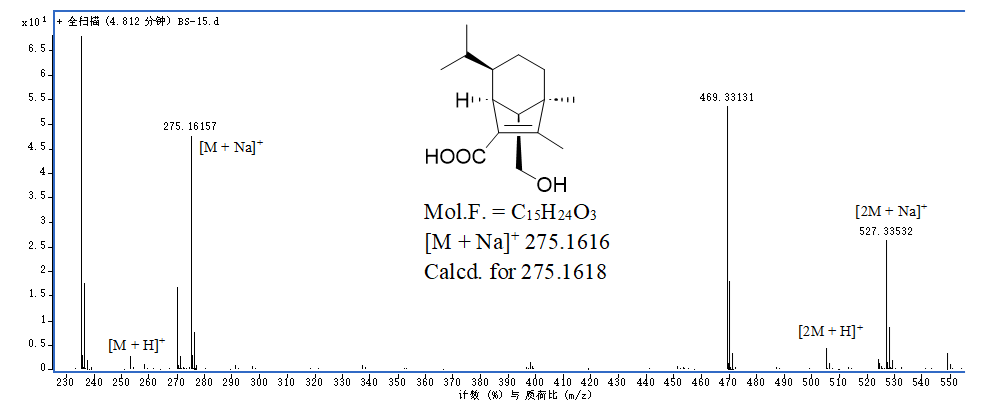
**

**Fig. S8a** HRESIMS spectrum of compound **8** in MeOH


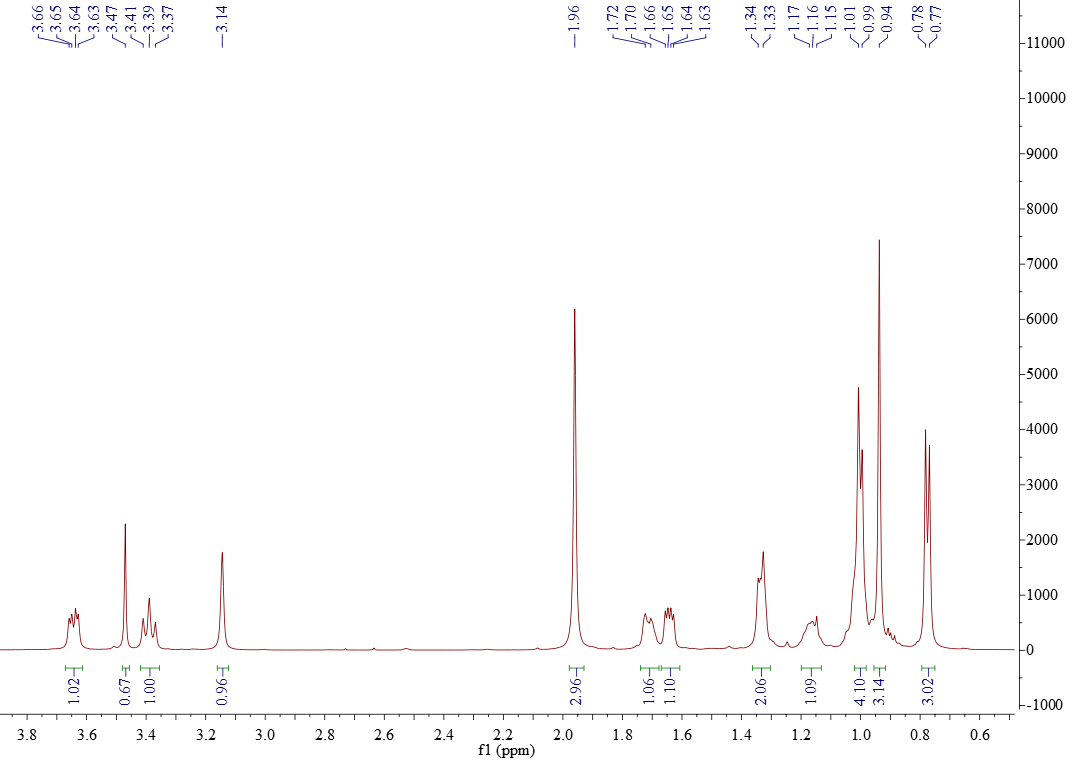


**Fig. S8b** ^1^H NMR (500 MHz, CDCl3) spectra of **8**


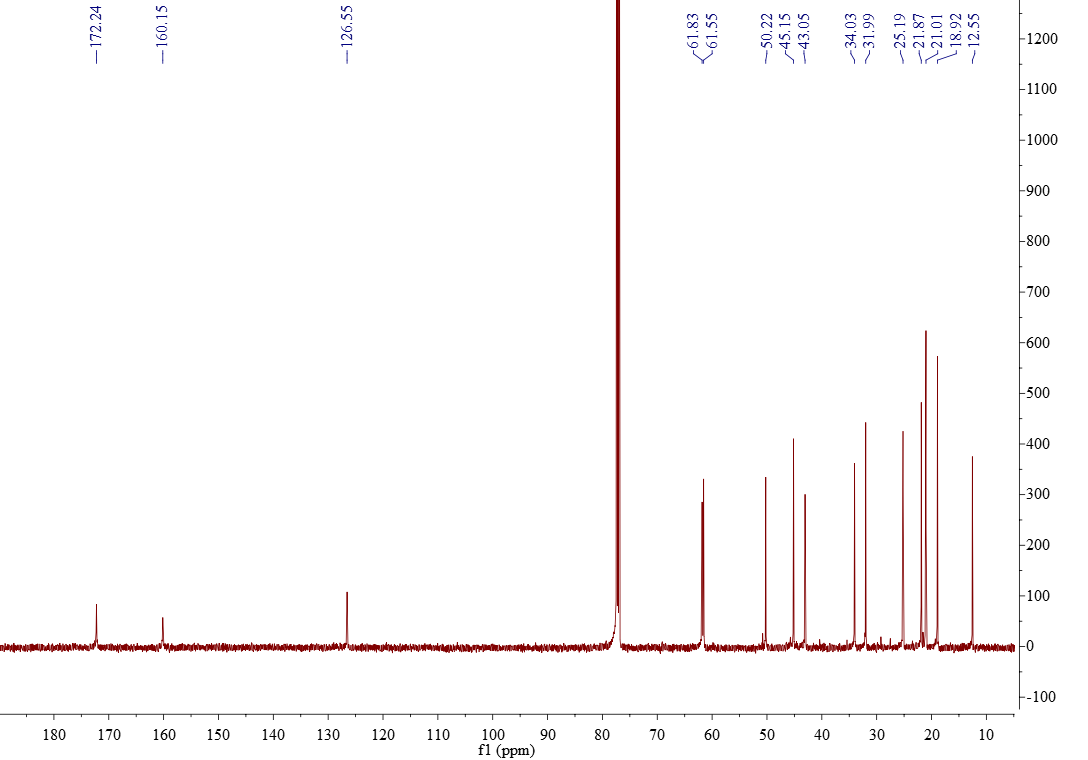


**Fig. S8c** ^13^C NMR (125 MHz, CDCl3) spectrum of **8**

**
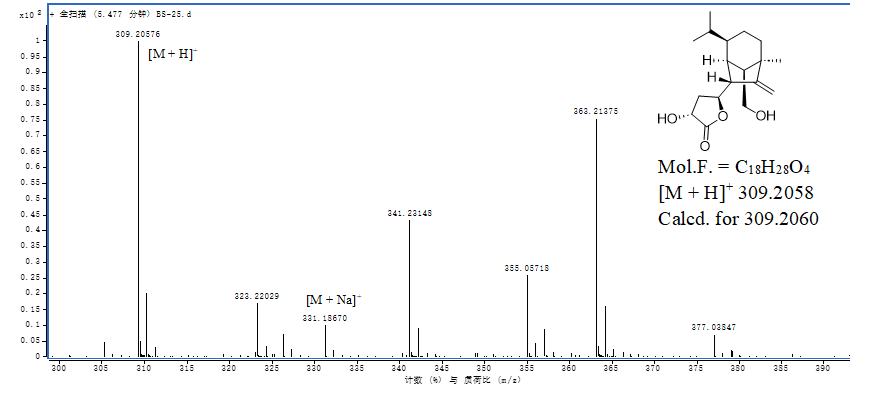
**

**Fig. S9a** HRESIMS spectrum of compound **9** in MeOH


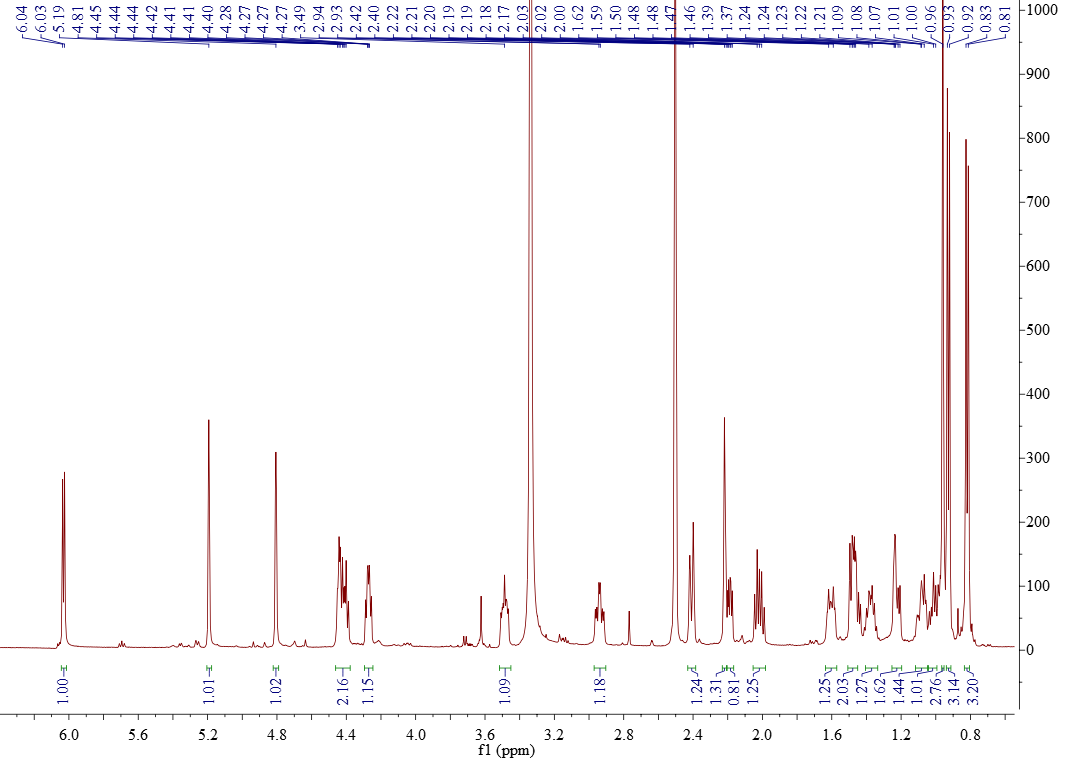


**Fig. S9b** ^1^H NMR (500 MHz, DMSO-*d*_6_) spectra of **9**


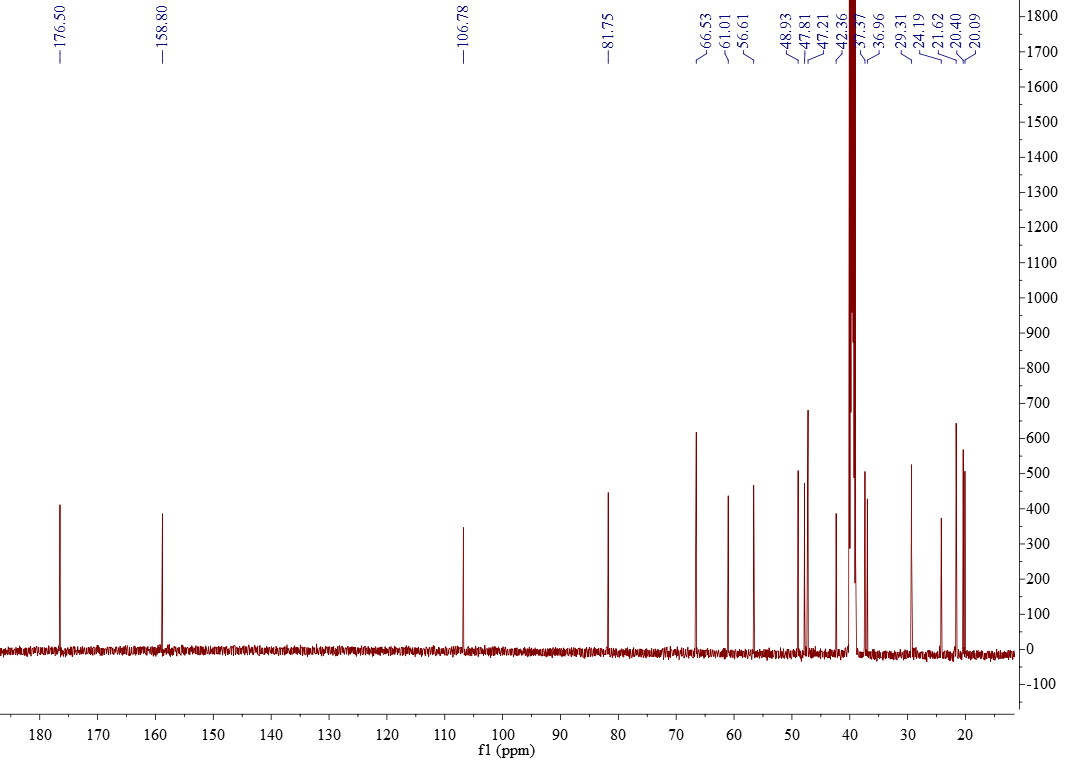


**Fig. S9c** ^13^C NMR (125 MHz, DMSO-*d*_6_) spectrum of **9**

**Table S7** ^1^H and ^13^C NMR Data of compounds **7**–**9**

|  | **7** | | **8** | | |  | **9** | |
| --- | --- | --- | --- | --- | --- | --- | --- | --- |
| Pos. | *δ*_H_^a^, mult (*J* in Hz) | *δ*_C_^b^ |  | *δ*_H_^a^, mult (*J* in Hz) | *δ*_C_^b^ |  | *δ*_H_^c^, mult (*J* in Hz) | *δ*_C_^d^ |
| 1 |  | 137.3 |  |  | 126.4 |  | 2.41 d (10.0) | 48.2 |
| 2 |  | 165.9 |  |  | 160.0 |  |  | 159.1 |
| 3 |  | 50.8 |  |  | 50.1 |  |  | 47.6 |
| 4 | 1.41 m | 34.2 |  | 1.41 m | 33.9 |  | 1.45 m  1.22 m | 42.8 |
| 5 | 1.75 m | 25.2 |  | 1.70 m | 25.1 |  | 1.60 m | 24.6 |
|  | 0.88 m |  |  |  |  |  | 1.00 m |  |
| 6 | 0.99 m | 44.9 |  | 0.99 m | 45.0 |  | 1.07 m | 49.4 |
| 7 | 3.19 brs | 41.3 |  | 3.15 brs | 42.9 |  | 2.21 brs | 37.8 |
| 8 | 1.04 s | 18.4 |  | 0.95 s | 18.8 |  | 0.96 s | 20.9 |
| 9 | 0.98 brs | 31.8 |  | 1.16 m | 31.9 |  | 1.37 m | 29.8 |
| 10 | 1.08 d (5.4) | 21.7 |  | 1.00 d (6.3) | 21.7 |  | 0.92 s | 22.1 |
| 11 | 0.77 d (5.4) | 20.7 |  | 0.78 d (6.3) | 20.9 |  | 0.82 s | 20.6 |
| 12 | 2.02 s | 10.6 |  | 1.97 s | 12.4 |  | 5.19 s | 107.1 |
|  |  |  |  |  |  |  | 4.81 s |  |
| 13 | 1.68 dd (8.7, 5.4) | 61.3 |  | 1.65 dd (9.4, 4.5) | 61.4 |  | 1.48 m | 56.8 |
| 14 | 3.65 dd (10.8, 5.4) | 62.5 |  | 3.65 dd (10.8, 4.5) | 61.7 |  | 3.50 m | 59.8 |
|  | 3.33 dd (10.8, 8.7) |  |  | 3.40 d (10.8) |  |  | 3.93 td (10.0, 5.0) |  |
| 15 | 10.00 s | 188.2 |  |  | 172.1 |  | 4.40 m | 82.3 |
| 1’ |  |  |  |  |  |  | 2.18 m | 37.4 |
|  |  |  |  |  |  |  | 2.01 m |  |
| 2’ |  |  |  |  |  |  | 4.27 m | 67.1 |
| 3’ |  |  |  |  |  |  |  | 177.1 |
| ^a^Recorded at 500 MHz in CDCl_3_. ^b^Recorded at 125 MHz in CDCl_3_. | | | | | | | | |
| ^c^Recorded at 500 MHz in DMSO-*d*_6_. ^d^Recorded at 125 MHz in DMSO-*d*_6_. | | | | | | | | |


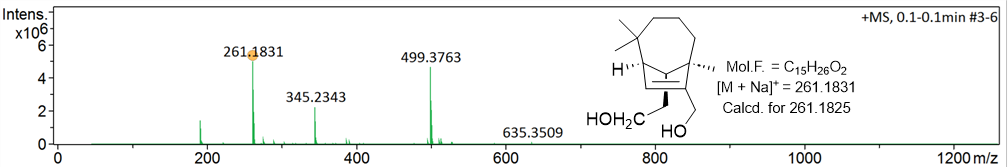


**Fig. S10a** HRESIMS spectrum of compound **10** in MeOH

**Fig. S10b** ^1^H NMR (800 MHz, DMSO-*d*_6_) spectra of compound **10**

**Fig. S10c** ^13^C NMR (200 MHz, DMSO-*d*_6_) spectrum of compound **10**

**Fig. S10d** HSQC (800 MHz, DMSO-*d*_6_) spectrum of compound **10**

**Fig. S10e** HMBC (800 MHz, DMSO-*d*_6_) spectrum of compound **10**

**Table S8** NMR data of compound **10**

| Position | *δ*_C_, mult | *δ*_H_, mult (*J* in Hz) |
| --- | --- | --- |
| 1 | 125.6, CH | 5.56 m |
| 2 | 147.2, C |  |
| 3 | 49.7, C |  |
| 4 | 44.0, CH_2_ | 1.61 m, 1.27 m |
| 5 | 20.7, CH_2_ | 1.47 m, 1.26 s |
| 6 | 41.2, CH_2_ | 1.30 m |
| 7 | 56.8, CH | 1.86 m |
| 8 | 21.1, CH_3_ | 0.92 s |
| 9 | 35.3, C |  |
| 10 | 27.4, CH_3_ | 0.87 s |
| 11 | 31.7, CH_3_ | 0.87 s |
| 12 | 57.7, CH_2_ | 3.87 m, 3.81 m |
| 13 | 45.2, CH | 1.86 m |
| 14 | 34.1, CH_2_ | 1.55 m, 1.10 m |
| 15 | 59.8, CH_2_ | 3.45 m, 3.34 m |

**Table S9.** DP4 probability of ^13^C NMR chemical shifts of **5a** (3*R**,6*S**,7*S**,9*S**,13*S**) and **5b** (3*R**,6*S**,7*S**,9*R**,13*S**).

| No. | *δ*exp | *δ*cal | | *δ*scal | | Corrected error (Δ*δ*) | | t distribution | | Probability | |
| --- | --- | --- | --- | --- | --- | --- | --- | --- | --- | --- | --- |
|  |  | **5a** | **5b** | **5a** | **5b** | **5a** | **5b** | **5a** | **5b** | **5a** | **5b** |
| 1 | 137.10 | 146.86 | 147.09 | 140.47 | 137.99 | -3.37 | -0.89 | 0.91 | 0.65 | 0.09 | 0.35 |
| 2 | 168.70 | 178.28 | 184.54 | 171.06 | 173.89 | -2.36 | -5.19 | 0.84 | 0.98 | 0.16 | 0.02 |
| 3 | 50.80 | 58.46 | 59.38 | 54.39 | 53.90 | -3.59 | -3.10 | 0.93 | 0.90 | 0.07 | 0.10 |
| 4 | 34.30 | 38.44 | 39.20 | 34.90 | 34.55 | -0.60 | -0.25 | 0.60 | 0.54 | 0.40 | 0.46 |
| 5 | 25.40 | 28.32 | 29.55 | 25.05 | 25.30 | 0.35 | 0.10 | 0.56 | 0.52 | 0.44 | 0.48 |
| 6 | 40.30 | 43.40 | 48.91 | 39.74 | 43.87 | 0.56 | -3.57 | 0.59 | 0.92 | 0.41 | 0.08 |
| 7 | 41.40 | 46.61 | 48.37 | 42.86 | 43.35 | -1.46 | -1.95 | 0.73 | 0.79 | 0.27 | 0.21 |
| 8 | 18.40 | 19.62 | 20.48 | 16.58 | 16.61 | 1.82 | 1.79 | 0.78 | 0.77 | 0.22 | 0.23 |
| 9 | 39.20 | 44.16 | 43.35 | 40.48 | 38.54 | -1.28 | 0.66 | 0.70 | 0.61 | 0.30 | 0.39 |
| 10 | 15.80 | 16.95 | 15.59 | 13.98 | 11.93 | 1.82 | 3.87 | 0.78 | 0.94 | 0.22 | 0.06 |
| 11 | 67.70 | 69.24 | 75.32 | 64.89 | 69.18 | 2.81 | -1.48 | 0.88 | 0.73 | 0.12 | 0.27 |
| 12 | 10.90 | 11.29 | 11.91 | 8.47 | 8.39 | 2.43 | 2.51 | 0.84 | 0.85 | 0.16 | 0.15 |
| 13 | 61.00 | 67.16 | 65.87 | 62.87 | 60.12 | -1.87 | 0.88 | 0.78 | 0.64 | 0.22 | 0.36 |
| 14 | 61.80 | 66.69 | 67.85 | 62.41 | 62.02 | -0.61 | -0.22 | 0.60 | 0.54 | 0.40 | 0.46 |
| 15 | 189.40 | 191.61 | 193.56 | 184.04 | 182.54 | 5.36 | 6.86 | 0.98 | 0.99 | 0.02 | 0.01 |
| Product of probabilities | | | | | | | | | | 1.02E-11 | 6.38E-13 |
| Bayes’s theorem probability (%) | | | | | | | | | | 94.10% | 5.90% |
